# The *Drosophila* CD36 Homologue *croquemort* Is Required to Maintain Immune and Gut Homeostasis during Development and Aging

**DOI:** 10.1371/journal.ppat.1005961

**Published:** 2016-10-25

**Authors:** Aurélien Guillou, Katia Troha, Hui Wang, Nathalie C. Franc, Nicolas Buchon

**Affiliations:** 1 Department of Entomology, Cornell University, Ithaca, NY, United States Of America; 2 Department of Cell & Molecular Biology, The Scripps Research Institute, La Jolla, CA, United States Of America; University of Michigan Medical School, UNITED STATES

## Abstract

Phagocytosis is an ancient mechanism central to both tissue homeostasis and immune defense. Both the identity of the receptors that mediate bacterial phagocytosis and the nature of the interactions between phagocytosis and other defense mechanisms remain elusive. Here, we report that Croquemort (Crq), a *Drosophila* member of the CD36 family of scavenger receptors, is required for microbial phagocytosis and efficient bacterial clearance. Flies mutant for *crq* are susceptible to environmental microbes during development and succumb to a variety of microbial infections as adults. Crq acts parallel to the Toll and Imd pathways to eliminate bacteria via phagocytosis. *crq* mutant flies exhibit enhanced and prolonged immune and cytokine induction accompanied by premature gut dysplasia and decreased lifespan. The chronic state of immune activation in *crq* mutant flies is further regulated by negative regulators of the Imd pathway. Altogether, our data demonstrate that Crq plays a key role in maintaining immune and organismal homeostasis.

## Author Summary

Phagocytosis is a first-line host defense mechanism against microbes. Interactions between phagocytosis and other immune mechanisms, such as the humoral response, however, remain elusive. Defective phagocytosis can lead to immune deficiencies and chronic auto-inflammation. Here, we show that Croquemort (Crq), a *Drosophila* member of the CD36 family of scavenger receptors, plays a role in microbial phagocytosis. Crq is required in phagocytes for efficient uptake of bacteria and fungi, and mutants for *crq* succumb to both environmental microbes and infections. Crq is also required for phagosome maturation, and *crq* mutants lack the ability to fully clear bacterial infection. As a result, *crq* mutant flies enter a state of chronic immune activation. Notably, they show increased production of the cytokine Upd3 that induces intestinal stem cell proliferation. Consequently, *crq* mutant flies show early signs of gut dysplasia, as well as a shortened lifespan. Altogether, our study demonstrates a new link between phagocytosis and tissue homeostasis, and illustrates how the chronic induction of cytokine production secondary to defective phagocytosis can alter gut homeostasis and shorten lifespan.

## Introduction

Mounting appropriate immune responses against pathogens is critical for the survival of all animals. Mechanisms to both eliminate microbes and resolve infection by returning the immune system to basal activity are necessary to maintain an adequate and balanced immune response [1,2]. Alterations in these responses can lead to immune deficiency or auto-inflammation [3–5]. Yet, to date, how these mechanisms are coordinated upon infection remains unclear.

*Drosophila* is a prime model to genetically dissect humoral and cellular innate immune responses to a variety of pathogens [6–8]. Humoral responses include the pro-phenoloxidase (PO) cascade, which leads to the generation of reactive oxygen species and melanization, and the rapid production of antimicrobial peptides (AMPs) regulated by the Toll and Imd pathways [7]. Upon recognition of microbial lysine (Lys)-type peptidoglycan (PGN), damage-associated molecular patterns (DAMPs), or exogenous protease activity, the Toll pathway promotes the nuclear translocation of the NF-κB-like transcription factor Dorsal-related Immune Factor (Dif) to induce AMP genes, such as *Drosomycin* [6,9]. In contrast, detection of bacterial meso-diaminopimelic acid (DAP)-type peptidoglycan activates the Imd pathway and leads to the nuclear translocation of the NF-κB-like transcription factor Relish (Rel) to induce transcription of AMP genes, such as *Diptericin* [10,11]. It has also been shown that proteases, such as Elastase and Mmp2, can activate the Imd pathway through cleavage of the receptor PGRP-LC [12]. As in mammals, chronic activation of immune responses is deleterious to the fly, and negative regulators are required to maintain immune homeostasis [13–15]. For instance, amidase PGN recognition proteins (PGRPs), such as PGRP-LB and PGRP-SC, negatively regulate the Imd pathway by enzymatically degrading PGN [14–16].

Phagocytosis and encapsulation are key cellular innate immune responses [7]. Phagocytosis allows for the uptake and digestion of microbes and apoptotic cells by phagocytes, including specialized immune cells called plasmatocytes [7,17]. Encapsulation results in the isolation and melanization of large materials, such as wasp eggs or damaged tissues, by dedicated immune cells named lamellocytes [18]. Both phagocytosis and humoral responses are required to fight infection. Indeed, decreasing the phagocytic ability of plasmatocytes by pre-injecting latex beads, which they take up, impairs fly survival upon infection with Gram-positive bacteria [19]. Similarly, inhibiting phagocytosis increases the susceptibility of Imd pathway-deficient flies to *Escherichia coli* (*E*. *coli*) infection, arguing that phagocytosis and the humoral response act in parallel [20]. Plasmatocytes were proposed to activate the production of AMPs by releasing immunostimulatory pathogen-associated molecular patterns (PAMPs) following phagocytosis [21]. They also express cytokines such as Unpaired 3 (Upd3), a ligand of the JAK-STAT pathway, which regulates immune-related genes [22]. Yet, ablation of the majority of plasmatocytes by targeted apoptosis has only a moderate effect on the fly’s ability to fight infection [23,24]. Therefore, the role of phagocytosis in the regulation of the humoral response and the resolution of infection remains unclear.

Several plasmatocyte receptors promote the recognition and engulfment of bacteria [25]. The scavenger receptor dSR-CI and a transmembrane protein, Eater, bind to both Gram-negative and -positive bacteria [26,27]. The membrane receptor PGRP-LC binds to and engulfs Gram-negative but not Gram-positive bacteria, and its membrane localization is dependent on the nonaspanin TM9SF4 [28,29]. Draper (Drpr) promotes clearance and degradation of neuronal debris and apoptotic cells via phagosome maturation, as well as phagocytosis of *Staphylococcus aureus (S*. *aureus)* together with the integrin βv and PGRP-SC1 [30–32]. Nimrod C1, which is related to Eater and Drpr, promotes phagocytosis of both *S*. *aureus* and *E*. *coli* by *Drosophila* S2 cells, and suppression of its expression in plasmatocytes inhibits phagocytosis of *S*. *aureus* [33]. Peste, a member of the CD36 family of scavenger receptors plays a role in the recognition and uptake of *Mycobacterium* by S2 cells [34]. Finally, *croquemort* (*crq*), another CD36 family member, promotes apoptotic cell clearance by embryonic plasmatocytes [35] and phagosome maturation of neuronal debris by epithelial cells [36].

In mammalian immunity, CD36 promotes the uptake of oxidized low density lipoproteins (oxLDLs) [37,38] and also regulates the host inflammatory response [39,40]. In addition, it is required to fight *Mycobacteria* and *S*. *aureus* infections in mice [41] and to induce pro-inflammatory cytokines in response to *Plasmodium falciparum* infection [42]. Using two lethal deficiencies that delete *crq* (as well as other genes), we previously proposed that Crq was specific to apoptotic cell clearance, as *crq*-deficient embryonic plasmatocytes retained some ability to engulf both *E*. *coli* and *S*. *aureus in vivo* [35]. However, Crq was subsequently implicated in phagocytosis of *S*. *aureus* by S2 cells, a heterogeneous cell line with phagocytic abilities derived from late embryonic stages [41]. Thus, we generated a knock-out of *crq* and further investigated its role in microbial phagocytosis and its relationship with the humoral response at larval and adult stages *in vivo*.

*Drosophila* plasmatocytes derive from pro-hemocytes originating either in the procephalic mesoderm of the embryo, with some further expanding by self-renewal in larval hematopoietic pockets, or from a second hematopoietic organ, the larval lymph glands, that persist to adulthood, or finally from adult hematopoietic hubs [43–51]. Here, we show that Crq is a major marker of plasmatocytes that is not required for hematopoiesis. The survival to adulthood of *crq* knock-out (*crq* ^*ko*^) mutants allowed us to quantitatively demonstrate that *crq* is required for pupae to survive environmental microbe infections and for adults to resist infection against Gram-negative and Gram-positive bacteria and fungi. *crq* ^*ko*^ flies tolerate infections as well as control flies, but are unable to efficiently eliminate microbes. Indeed, *crq* ^*ko*^ plasmatocytes are poorly phagocytic and defective in phagosome maturation. Crq acts parallel to the Imd and Toll pathways in eliminating pathogens, and *crq* ^*ko*^ flies display elevated and persistent *Dpt* and *upd3* expression, demonstrating that mutating *crq* promotes a state of chronic immune activation. As a consequence, *crq* ^*ko*^ flies die prematurely with early signs of gut dysplasia and premature intestinal stem cell hyperproliferation. Therefore, we propose a model wherein *crq* is central to immune and organismal homeostasis. Overall, our results shed new light on the links between phagocytes, commensal microbes, gut homeostasis, and host lifespan.

## Results

### Croquemort is a major plasmatocyte marker and not required for hematopoiesis

**Fig 1:**
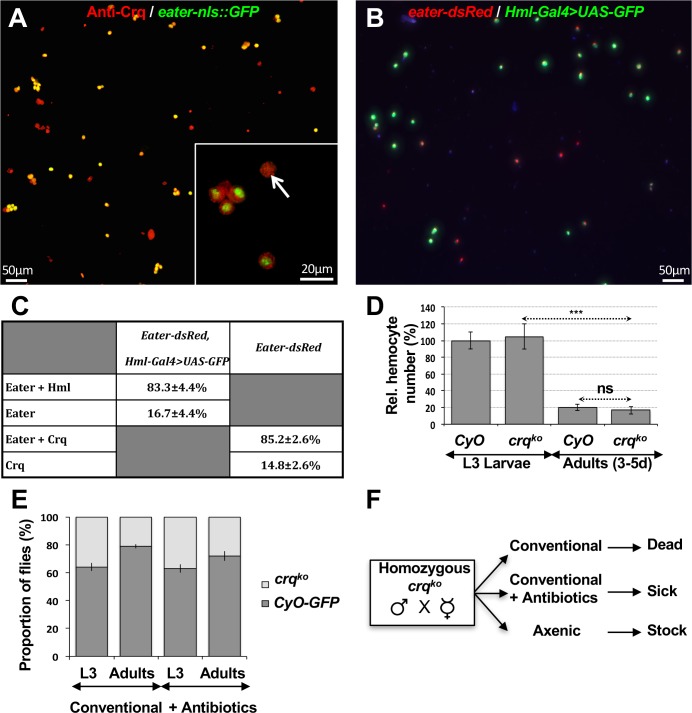
Crq is a major plasmatocyte marker that is required for survival to environmental microbes during pupariation. (**A-B**) Crq and GFP immunostainings of *eater-nlsGFP* (**A**) and GFP immunostaining of *eater-dsRed*; *hml-gal4>UAS-GFP* plasmatocytes (**B**). (**C**) Quantification of experiments in **A** and **B** reveals subpopulations of Crq-positive plasmatocytes, of Crq- and Eater-positive plasmatocytes, and of a majority of plasmatocytes expressing all three markers Crq, Eater and HML. (**D**) Relative hemocyte numbers (in %) of *crq* ^*ko*^ larvae and 3-to-5- day-old adults compared to wild-type controls. Mean values of at least 5 repeats are represented ±SE. ***p<0.001 (Student’s T-test). (**E**) Percentages of homozygous *crq* ^*ko*^ versus CyO-GFP-positive L3 larvae or adult flies emerging from *crq* ^*ko*^ */CyO*,*GFP* heterozygous stock maintained on conventional or antibiotic-supplemented medium. (**F**) Schematic of health status of *crq* ^*ko*^ homozygous individuals emerging from cross of *crq* ^*ko*^ homozygous males and females on conventional, antibiotic-supplemented or axenic medium.

In *Drosophila* adults, plasmatocytes (the phagocytic hemocyte lineage) originate from both embryonic and larval hematopoiesis [52]. *crq* is expressed in embryonic and larval plasmatocytes, as well as in S2 cells [53]. To test whether *crq* is expressed in adult plasmatocytes, we performed dual staining with combinations of GFP or dsRed and Crq antibodies of hemocytes bled from previously characterized transgenic plasmatocyte-reporter lines: *eater-nls*::*GFP*, *eater-dsRed*, and *Hml-Gal4>UAS-GFP* (Hemolectin-positive hemocytes) [54,55] (**Fig 1A and 1B**). We found that 83.3±4.4% of hemocytes of *Hml-Gal4>UAS-GFP* and *eater-dsRed* carrying flies were positive for both markers, while 16.7±4.4% were positive for *eater-dsRed* alone (**Fig 1C**). Crq immunostaining of hemocytes bled from *eater-nls*::*GFP* flies revealed that 85.2±2.6% of them were Crq and eater-dsRed positive, while 14.8±2.6% were Crq-positive but did not express eater-dsRed (**Fig 1C**). From this, we extrapolated that about 72.4% of circulating hemocytes are positive for all three markers, 12.8% are double positive for Crq and Eater, and 14.8% solely express Crq (**Fig 1C**). Therefore, *crq* is expressed in all Eater and Hml-positive hemocytes and marks the majority, if not all, adult plasmatocytes.

To study its role *in vivo*, we generated a knock-out allele of *crq* (*crq* ^*ko*^) by homologous recombination [36]. This mutant deletes the entire *crq* open reading frame (**S1A Fig**), and thus abolishes its expression [36]. As previously reported [35], *crq* was not required for embryonic hematopoiesis. As for *crq* deletion mutants, *crq* ^*ko*^ embryonic plasmatocytes were less efficient at clearing apoptotic cells, having a phagocytic index of 1.6±0.2 versus 2.45±0.3 apoptotic cells/plasmatocyte for wild-type embryos (p<0.05, **S1B Fig**). Homozygous *crq* ^*ko*^ flies were viable and appeared morphologically normal. To ask whether *crq* is required for hematopoiesis at later developmental stages, we recombined an *eater-nls*::*GFP* transgene (i.e., the broadest plasmatocyte reporter after Crq) (**Fig 1C**) into the *crq* ^*ko*^ mutants, bled larvae and adults, and semi-automatically scored their *eater-nls*::*GFP* positive plasmatocytes by microscopy (**S1C Fig** and **Fig 1D**). As previously reported for wild-type [56,57], adult *crq* ^*ko*^ flies had about 5-fold less plasmatocytes than larvae, and their number of *eater-nls*::*GFP*-positive plasmatocytes at both larval and adult stages were similar to that of wild-type flies (**Fig 1D**). Pro-hemocytes that differentiate into plasmatocytes can also differentiate into crystal cells, which are involved in melanization [58]. Furthermore, self-renewing plasmatocytes of the embryonic lineage can also differentiate into crystal cells by trans-differentiation [59,60]. Thus, we tested whether *crq* ^*ko*^ flies have differentiated crystal cells by scoring the melanotic dots formed following heat-induced crystal cell lysis. We found no significant difference between *crq* ^*ko*^ and wild-type larvae (**S1D Fig**). Therefore, Crq is a major plasmatocyte marker that is not required for hematopoiesis or hemocyte differentiation.

### *croquemort* mutant flies are susceptible to environmental microbes

While *crq* ^*ko*^ homozygous flies were viable to adulthood, we could not maintain a homozygous stock on conventional fly food. We found that 36±3.2% homozygous *crq* ^*ko*^ larvae arose from crosses between *crq* ^*ko*^ heterozygous flies over GFP-marked CyO balancer chromosome, indicating full viability of the homozygous larvae (**Fig 1E**). However, only 18±1.7% of emerging adults were homozygous *crq* ^*ko*^ flies, indicating that half of the *crq* ^*ko*^ homozygous progeny died during pupariation. Because flies with decreased plasmatocyte counts undergo pupal death associated with the presence of otherwise innocuous environmental microbes [23], we asked whether supplementing the food with antibiotics could rescue *crq* ^*ko*^ lethality. With this treatment, we recovered 29±3.6% of *crq* ^*ko*^ homozygous adults (**Fig 1E),** indicating a partial rescue of pupal lethality (homozygous vs balanced adults, p = 0.021). These results suggest that *crq* ^*ko*^ pupae are susceptible to environmental microbes.

No adult progeny could be recovered from *crq* ^*ko*^ homozygous crosses on conventional fly food, but *crq* ^*ko*^ adults emerged in the presence of antibiotics that gave rise to a second adult progeny (**Fig 1E and 1F**). Maintaining a homozygous viable stock with antibiotics, however, remained difficult. We next bleached homozygous *crq* ^*ko*^ embryos and raised them on sterile food. Under these axenic conditions, we successfully cultured a homozygous *crq* ^*ko*^ line (**Fig 1F**). Therefore, environmental microbes represent a health constraint for *crq* ^*ko*^ homozygous flies.

### *croquemort* mutant flies are broadly susceptible to infection

**Fig 2:**
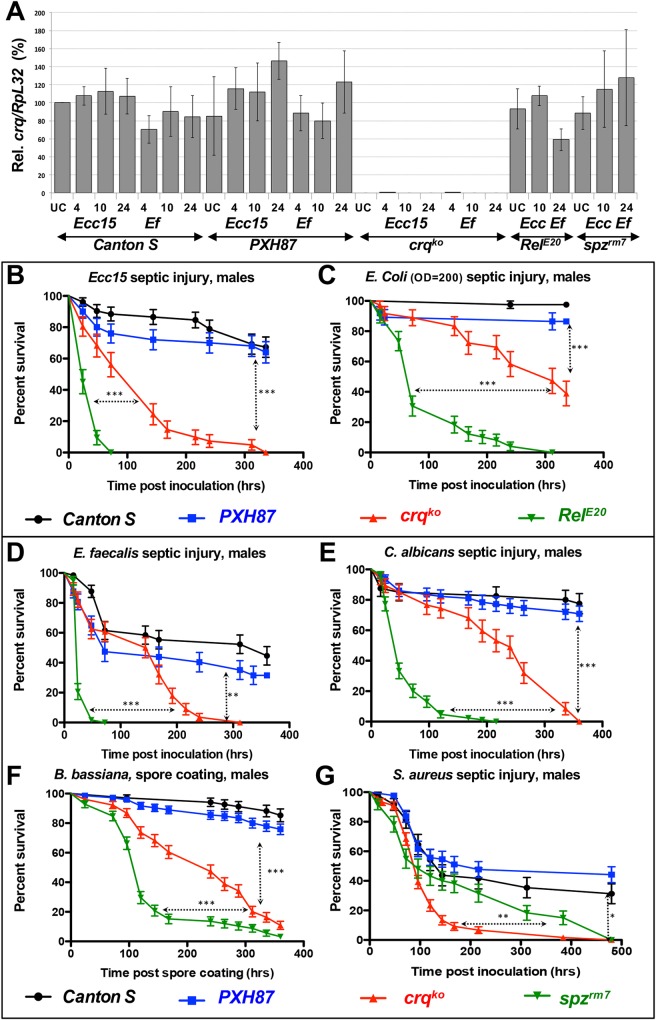
*crq* knock-out flies are broadly susceptible to infection. (**A**) Relative percentages of *crq* mRNA levels of UC *Cs* and *PXH87* controls, *crq* ^*ko*^, *Rel* ^*E20*^, and *spz* ^*rm7*^ mutant flies at 4, 10 or 24 hrs after *Ecc15* or *E*. *faecalis* infections when compared to that of UC *Cs* flies. Mean values of at least 3 repeats are represented ± SE. (**B**-**G**) Survival curves (in %) over time of *Cs* and *PXH87* control flies, *crq* ^*ko*^, and *Rel* ^*E20*^ or *spz* ^*rm7*^ homozygous male flies after septic injury with *Ecc15* (**B**), *E*. *coli* (**C**), *E*. *faecalis* (**D**), *C*. *albicans* (**E**), *S*. *aureus* (**G**), or after spore coating with *B*. *bassiana* (**F**). The curves represent the average percent survival ±SE. **p<0.01 ***p<0.001 in a log rank test.

The susceptibility of *crq* ^*ko*^ pupae to environmental microbes suggested that *crq* is required to mount an appropriate immune response. We next asked whether *crq* was up-regulated in flies injected with the Gram-negative bacterium *Pectinobacterium* (previously known as *Erwinia) carotovora 15* (*Ecc15*) or the Gram-positive *Enterococcus faecalis* (*E*. *faecalis*). As anticipated, there was no *crq* expression in unchallenged (UC) or infected *crq* ^*ko*^ flies as detected by RT-qPCR (**Fig 2A**). While *crq* was expressed in both UC *pXH87-crq* transgenic (the parental transgenic strain used for the generation of *crq* ^*ko*^ flies, hereafter referred to as *PXH87*) and *Canton S (Cs)* control flies, it was not up-regulated within the first 24hrs of infection with *Ecc15* or *E*. *faecalis* (**Fig 2A**). However, we cannot exclude the possibility that *crq* may be up-regulated in plasmatocytes specifically at these early time points after infection. Its expression was also not altered in mutant flies for the NF-κB-like transcription factor *Relish* (*Rel* ^*E20*^ *)* downstream of the Imd pathway, or in flies mutant for the Toll ligand *spz (spz* ^*rm7*^ *)*, upstream of the Toll pathway during that time-frame [9]. Surprisingly, we did observe an increase in *crq* mRNA levels at 36 (p = 0.0076) and 132 hrs (p = 0.0213) post *Ecc15* infection (**S2A Fig**), but did not detect any upregulation of *crq* mRNA levels at 36 and 132 hours post *E*. *faecalis* infection (**S2B Fig**) (p>0.05). Altogether, our data show that *crq* does not appear to be induced by infection in whole adult extracts during the first 24 hours post infection with *Ecc15* and *E*. *faecalis*, and its expression appears independent of the Toll and Imd pathways. However, at later time-points after infection *crq* can be upregulated in a pathogen-specific manner, as seen with *Ecc15* here.

To assess the susceptibility of *crq* ^*ko*^ male (**Fig 2B–2G**) and female (**S2B–S2G Fig**) flies to a variety of pathogens, we monitored their survival to these infections over time. When challenged by septic injury with the Gram-negative bacterium *Ecc15*, male (**Fig 2B**) and female (**S2C Fig**) *crq* ^*ko*^ flies were more susceptible than *Cs* and *PXH87* control flies to this infection (p<0.0001). *crq* ^*ko*^ flies all died within 336 hrs post-infection (hpi), while only 64±6.8% and 67±6.5% of *PXH87* and *C*s flies had died by that time-point. *crq* ^*ko*^ flies were, however, less susceptible than *Rel* ^*E20*^ mutants (p<0.0001), which are defective in the production of AMPs downstream of Imd [61]. All *Rel* ^*E20*^ flies died within 72 hpi, while only 56±7.7% of *crq* ^*ko*^ flies had succumbed by that same time-point (**Fig 2B**). To verify that the susceptibility to *Ecc15* infection was due to the *crq* ^*ko*^ mutation and not to a background mutation, we infected trans-heterozygous flies for *crq* ^*ko*^ and *Df(2L)BSC16*, which deletes *crq*, with *Ecc15* (**S2D Fig**). These flies were as susceptible to *Ecc15* infection as the *crq* ^*ko*^ homozygous flies; they all died within 288 hpi, indicating that the *crq* mutation is responsible for this phenotype (**S2D Fig**). *crq* ^*ko*^ flies also succumbed to infection with *E*. *coli* (39±8.1% survival at 336 hpi), a Gram-negative bacterium that does not kill *Cs* (97±2.5% survival) or *PXH87* (86±5.6% survival) flies. However, *crq* ^*ko*^ flies were less susceptible to *E*. *coli* infection than *Rel* ^*E20*^ flies, which all died within 312 hpi (p<0.0001) (**Fig 2C** and **S2E Fig**). Therefore, *crq* ^*ko*^ flies are susceptible to various Gram-negative bacterial infections.

Similarly, *crq* ^*ko*^ flies were more susceptible to infection with the Gram-positive bacterium *E*. *faecalis* than controls (p = 0.0006) (**Fig 2D** and **S2F Fig**) and died in 312 hpi. However, they were less susceptible than *spz* ^*rm7*^ flies (p<0.0001), which are defective in the production of AMPs downstream of Toll and died within 72 hpi (**Fig 2D** and **S2F Fig**). *crq* ^*ko*^ flies also died with intermediate susceptibility between that of control and *spz* ^*rm7*^ flies (p<0.0001 for both) after septic injury with the pathogenic yeast *Candida albicans* (**Fig 2E** and **S2G Fig).** Similarly, *crq* ^*ko*^ flies were significantly more susceptible to exposure to spores of the entomopathogenic fungus *Beauveria bassiana* than *Cs* and *PXH87* flies (p<0.0001), but less susceptible than *spz* ^*rm7*^ flies (p<0.0001) (**Fig 2F** and **S2H Fig**). Finally, *crq* ^*ko*^ flies were more susceptible to *S*. *aureus* infection than *spz* ^*rm7*^ flies (p = 0.0073) (**Fig 2G** and **S2I Fig**), and *spz* ^*rm7*^ flies were only slightly more susceptible than *Cs* and *PXH87* flies (p = 0.0006 and p<0.0001 respectively). Therefore, *crq* ^*ko*^ flies are susceptible to Gram-positive bacteria and fungal infections and strongly susceptible to infection with *S*. *aureus*, a bacterium specifically cleared by phagocytosis [19,62,63].

These results argue that *crq* is required to fight infection. To further confirm this, we drove the expression of a *UAS-crq* transgene under the control of a *crq* promoter-Gal4 driver in the *crq* ^*ko*^ flies (*crq* ^*ko*^ *; crq-Gal4>UAS-crq*). These rescue flies were no longer susceptible to *Ecc15* (**S3A Fig**), *E*. *faecalis* (**S3B Fig**), and *B*. *bassiana* (**S3C Fig**) infections (non-significant (ns) compared to *PXH87*, and p<0.0001 when compared to *crq* ^*ko*^ flies) (**S3A–S3C Fig**). To assess the possible requirement of *crq* in hemocytes, we drove the expression of a *UAS-crq* transgene under the control of a hemocyte-specific *serpent* promoter-Gal4 driver in the *crq* ^*ko*^ flies (*crq* ^*ko*^ *; srp-Gal4>UAS-crq*). These flies were significantly less susceptible to *Ecc15*, *E*. *coli*, *E*. *faecalis* and *C*. *albicans* infections than *crq* ^*ko*^ flies (p<0.0001, p<0.0001, p = 0.0004 and p<0.0001, respectively) (**S3D–S3G Fig**). We did not observe any significant differences between rescue experiments with the *crq-Gal4* or *srp-Gal4* drivers after infection with *Ecc15*, *E*. *coli*, or *E*. *faecalis (p>0*.*05)*. The hemocyte-specific rescue of *crq* ^*ko*^ flies infected with *C*. *albicans*, however, was slightly less efficient than the rescue with the *crq-Gal4* driver (p = 0.0269). Thus, *crq* appears to be required mostly in phagocytes to fight infection by both Gram negative and Gram positive bacteria, although it appears to also be required in other tissues to fight *C*. *albicans* infection.

### *croquemort* mutant flies are tolerant but poorly resistant to infection

Multi-cellular organisms use two complementary strategies to fight infection: resistance, to eliminate microbes, and tolerance, to allow them to endure the infection and/or its deleterious effects [64,65]. Compared to controls, *crq* ^*ko*^ flies die prematurely at around 552 hours even in the absence of infection (**S4A Fig**), suggesting these flies could be generally unfit or susceptible to damage. To test their response to abiotic damage, we pricked *crq* ^*ko*^ flies with sterile needles at two separate thoracic sites. These flies did not die any earlier than non-pricked *crq* ^*ko*^ flies (**S4A Fig**). Thus, despite their decreased lifespan, *crq* ^*ko*^ flies are not susceptible to aseptic wounds.

**Fig 3:**
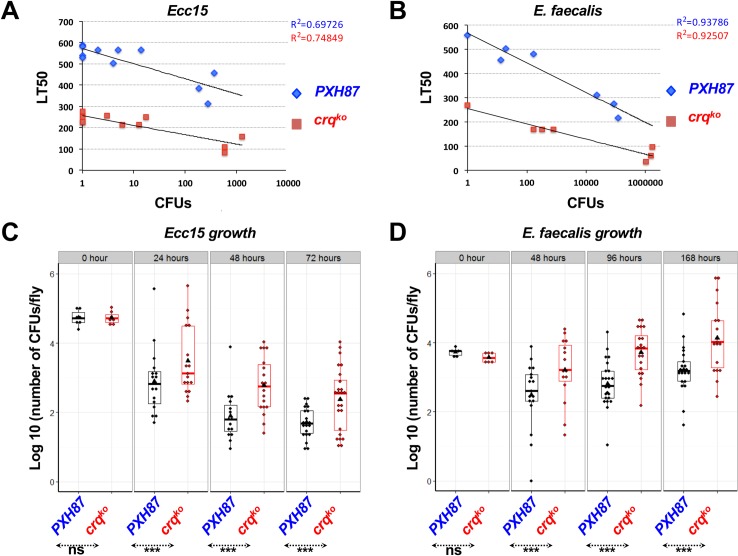
*crq* knock-out flies are less resistant to infection than wild-type but equally tolerant. (**A**-**B**) Tolerance graphs given as the plot of regression between LT50 and the number of CFUs of *Ecc15* (**A**) or *E*. *faecalis* (**B**) found in flies at 24hrs after septic injury for *crq* ^*ko*^ homozygous and *PXH87* flies. (**C**-**D**) Resistance graphs given as the log number of CFUs of *Ecc15* (**C**) or *E*. *faecalis* (**D**) per *crq* ^*ko*^ homozygous and *PXH87* flies over time after septic injury. **p<0.01 ***p<0.001.

To date, few studies have quantified the tolerance of immune-deficient flies [66,67]. Tolerance can be measured as the dose response curve relating health to microbe load. This curve takes the shape of a sigmoid; life expectancy in unchallenged conditions is considered as vigor, and the slope of the response curve (the portion of the health/load curve which is linear) estimates the ability to tolerate infection (**S4B Fig**) [67]. *crq* ^*ko*^ flies have shortened lifespan and therefore an altered vigor (**S4A Fig**). We further aimed to estimate whether *crq* ^*ko*^ flies show a decrease in tolerance by measuring the relationship (statistical interaction) between microbial load and the corresponding health of the host [64,67]. We used three approximations to relate health to microbe load of *crq* ^*ko*^ flies and focused on the linear part for each regression. First, we estimated the regression between the LT50 (time at which 50% of the flies are dead) of *Ecc15* or *E*. *faecalis*-infected flies and the number of bacteria injected (measured as colony forming units or CFUs) (**S4C and S4D Fig**). We did not detect any significant LT50~Time interaction between *PXH87* and *crq* ^*ko*^ flies (p = 0.21782 for *E*. *faecalis*, p = 0.55800 for *Ecc15*) (**S4C and S4D Fig**). However, this measure of bacterial load does not take into account the growth of the pathogen within the host. We therefore also quantified the regression between LT50 and the number of bacteria in the flies at 24 hpi (**Fig 3A and 3B**). We detected significant LT50~Time interaction between *PXH87* and *crq* ^*ko*^ flies (p = 0.008486 for *E*. *faecalis*, p = 0.018965 for *Ecc15*), with PXH87 flies having lower tolerance than *crq* ^*ko*^ flies (**Fig 3A and 3B**). Finally, to get another estimate of the health of the flies, we plotted the health/bacterial load curve using survival at 3 time-points post *Ecc15* infection and their corresponding bacterial load (**S4E Fig**). We did not detect any significant survival-time interaction between *PXH87* and *crq* ^*ko*^ flies (p = 0.335111). Thus, while *crq* ^*ko*^ flies die prematurely in the absence of infection, they do not show any decreased tolerance to infection when compared to control flies.

These data suggest that the increased susceptibility of *crq* ^*ko*^ flies to infection is due to their inability to control bacterial growth. In order to test this hypothesis, we monitored bacterial load during the course of *Ecc15* and *E*. *faecalis* infections. In *PXH87* flies, *Ecc15* is eliminated within the first 48hrs of infection to reach an apparent plateau of low number of CFUs that persist at 72hrs post-infection (**Fig 3C**). *crq* ^*ko*^ flies were less able to clear *Ecc15* than controls with higher bacterial loads throughout the infection (p<0.001 for 24, 48 and 72hrs) (**Fig 3C**). In contrast, despite an initial decline of CFUs at 48hrs, *E*. *faecalis* grew within control flies at 96 and 168hrs (**Fig 3D**). During the whole course of infection with *E*. *faecalis*, the bacterial loads were significantly lower in wild-type control flies than in the *crq* ^*ko*^ flies (p<0.001 at 48, 96 and 168hrs) (**Fig 3D**). These data indicate that *crq* is required for efficient elimination of both *Ecc15* and *E*. *faecalis*.

### *croquemort* is required for engulfment of bacteria and phagosome maturation

**Fig 4:**
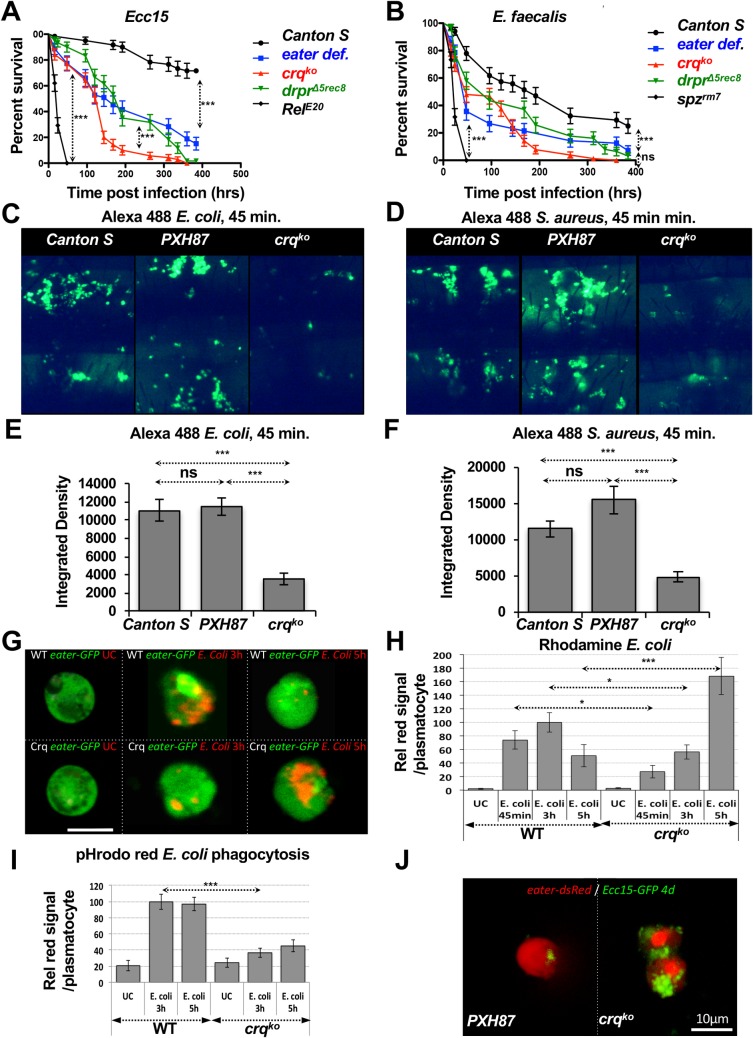
*crq* is required for efficient phagocytosis of bacteria and phagosome maturation. (**A**-**B**) Survival curves (in %) over time of *Canton S*, *crq* ^*ko*^, *drpr* ^*Δ5rec8*^, *eater* deficient flies, and *spz* ^*rm7*^ or *Rel* ^*E20*^ mutant flies after septic injury with *Ecc15* (**A**) and *E*. *faecalis* (**B**). (**C**-**D**) Representative fluorescent images of abdomen sections of *Canton S*, *PXH87*, *crq* ^*ko*^ mutant and *crq* ^*ko*^; *crq-gal4>UAS-eGFP* rescue flies at 45min after injection of Alexa 488-labeled *E*. *coli* (**C**) and *S*. *aureus* (**D**). (**E**-**F**) Quantifications of Alexa 488 fluorescent integrated density (IntDen) of experiments highlighted in **C** and **D**. respectively. (**G**) Confocal micrographs of plasmatocytes from wild-type and *crq* ^*ko*^ homozygous flies carrying the *eater-GFP* transgene (in green) bled either before (UC) or at 3 and 5hrs after injection with Rhodamine-labeled *E*. *coli* (in red). Scale bar, 10μm. (**H**) Quantification of the experiment in (**G**) given as the percentage of Rhodamine fluorescence of bacteria contained within plasmatocytes relative to control plasmatocytes bled at 3hrs after challenge of wild-type and *crq* ^*ko*^ homozygous flies. Results are presented for UC flies and flies at 45 min, 3 and 5hrs post-injection with Rhodamine *E*. *coli*. (**I**) Same quantification as in (**H**) for experiment with pHrodo red-labeled *E*. *coli*. (**J**) Confocal micrographs of plasmatocytes (in red) bled from *PXH87* control and *crq* ^*ko*^ flies carrying the *eater*::*dsred* transgene 4 days post-injury with GFP-expressing *Ecc15*.

*crq* is required for efficient phagocytosis of apoptotic cells (also known as efferocytosis) *in vivo*, and phagocytosis of *S*. *aureus* by S2 cells (**S1B Fig** and [35,41]). In addition, rescue of *crq* expression in hemocytes improved survival to various infections (**S3D–S3G Fig**), suggesting that *crq* could alter microbial phagocytosis. To test this hypothesis, we first compared the susceptibility of *crq* ^*ko*^ flies to infection with that of mutants for two phagocytic receptors, Eater and Drpr [26,31]. *crq* ^*ko*^ flies succumbed to *Ecc15* infection significantly faster than *eater-*deficient (p = 0.0002) and *drpr* ^*rec8Δ5*^ loss-of-function flies (p<0.0001). 90±3.58% of *crq* ^*ko*^ flies died within 192 hpi, while only 60±6.77% of *drpr* and *eater* mutants died in that same time (**Fig 4A**). However, the *crq* ^*ko*^ flies were significantly less susceptible to *Ecc15* than *Rel* ^*E20*^ flies (p<0.0001), which all died within 48 hpi (**Fig 4A**). In contrast, *crq* ^*ko*^ flies succumbed to *E*. *faecalis* infection at a similar pace to that of both *eater*-deficient and *drpr* ^*rec8Δ5*^ flies with 80–90% of all strains dying within 240 hpi (**Fig 4B**). However, all mutants were significantly less susceptible than *spz* ^*rm7*^ flies, which died within 48 hrs of *E*. *faecalis* infection (p<0.0001) (**Fig 4B**).

To examine the precise role of *crq* in phagocytosis, we compared the amount of bacteria engulfed within 45min of thoracic injections of dead, Alexa 480-labeled *E*. *coli* and *S*. *aureus* in *Cs*, *PXH87*, and *crq* ^*ko*^ flies as previously described [20,26] (**Fig 4C and 4D**). The *crq* ^*ko*^ flies engulfed both *E*. *coli* and *S*. *aureus* bacteria with on average 66% less efficiency than control flies (**Fig 4C–4F**, respectively). This phenotype was completely rescued in *crq* ^*ko*^ flies expressing a *UAS-crq* transgene under a *crq-Gal4* driver (*crq* ^*ko*^, *crq-Gal4>UAS-crq*), which appeared to engulf more efficiently than control *PXH87* flies (**S5A Fig** and **S5B Fig**). We speculate that this difference was due to the overexpression of *crq* in those flies.

To further assess the phagocytosis phenotype, wild-type and *crq* ^*ko*^ flies carrying the *eater-nls*::*GFP* plasmatocyte-reporter were injected with dead rhodamine-labeled *E*. *coli*, bled, and their plasmatocytes were analyzed by confocal microscopy for internalized bacteria (**Fig 4G**). The rhodamine-fluorescence per *eater-nls*::*GFP* plasmatocyte was quantified at 45min, 3hrs, and 5hrs post-injection and normalized to that of WT plasmatocytes at 3hrs post-injection (**Fig 4H**). In WT plasmatocytes, the relative rhodamine-fluorescence increased as early as 45min, peaked at 3hrs, and decreased after 5hrs, as bacteria were presumably digested in mature phagosomes (**Fig 4H**). In contrast, *crq* ^*ko*^ plasmatocytes accumulated about 2-fold fewer bacteria than controls at 45min and 3hrs post-injection, but accumulated 1.7-fold more bacteria by 5hrs post-injection. In addition, at 45min post-injection, most bacteria were internalized within wild-type plasmatocytes (**S5C Fig**), whereas bacteria were often bound to the cell surface of *crq* ^*ko*^ plasmatocytes without being internalized (**S5D Fig**). Thus, *crq* ^*ko*^ plasmatocytes can engulf bacteria but are less efficient at it than controls at early time-points; they also appear to accumulate internalized bacteria over time. These results are consistent with a role for *crq* in promoting efficient uptake of bacteria. Moreover, the observed accumulation of bacteria in *crq* ^*ko*^ plasmatocytes at 5hrs post-injection suggested that *crq* could also be required for phagosome maturation and digestion of bacteria. To test this, we injected control, c*rq* ^*ko*^, and rescue flies with pH-sensitive pHrodo *E*. *coli* and *S*. *aureus*. pHrodo bacteria fluoresce when engulfed into a fully mature, acidified phagosome [68] (**S5E and S5F Fig)**. After quantification, we observed about 50% less fluorescence in *crq* ^*ko*^ when compared to controls at 1, 3, and 5hrs post-injection (**S5G and S5H Fig**, p<0.5 when comparing *PXH87* and *crq* ^*ko*^ flies). This phenotype was again completely rescued in *crq* ^*ko*^, *crq-Gal4>UAS-crq* flies (**S5E and S5F Fig** and **S5G and S5H Fig**, p>0.5 when comparing *PXH87* and rescue flies). At the single cell level, *crq* ^*ko*^ plasmatocytes had up to 63±5.66% and 55±7.46% less pHrodo *E*. *coli* than controls at 3 and 5hrs, respectively (**Fig 4I**).

Finally, to ask whether mutating *crq* resulted in persistence of pathogenic bacteria, we injected live GFP-labeled *Ecc15* in control and *crq* ^*ko*^ flies carrying the *eater-dsred* plasmatocyte reporter (**Fig 4J** and **S5I Fig**). Control *PXH87* plasmatocytes had little to no GFP signal at 4 days post-infection, indicating that most bacteria had been engulfed and digested (**Fig 4J** and **S5I Fig**). In contrast, *crq* ^*ko*^ plasmatocytes had a 6-fold higher GFP signal, demonstrating that live *Ecc15* accumulate in *crq* ^*ko*^ plasmatocytes (**Fig 4J** and **S5I Fig**). Taken together, these results show that *crq* is required for efficient microbial phagocytosis by playing a role in bacterial uptake and phagosome maturation.

### Croquemort acts in parallel to the Imd and Toll pathways

**Fig 5:**
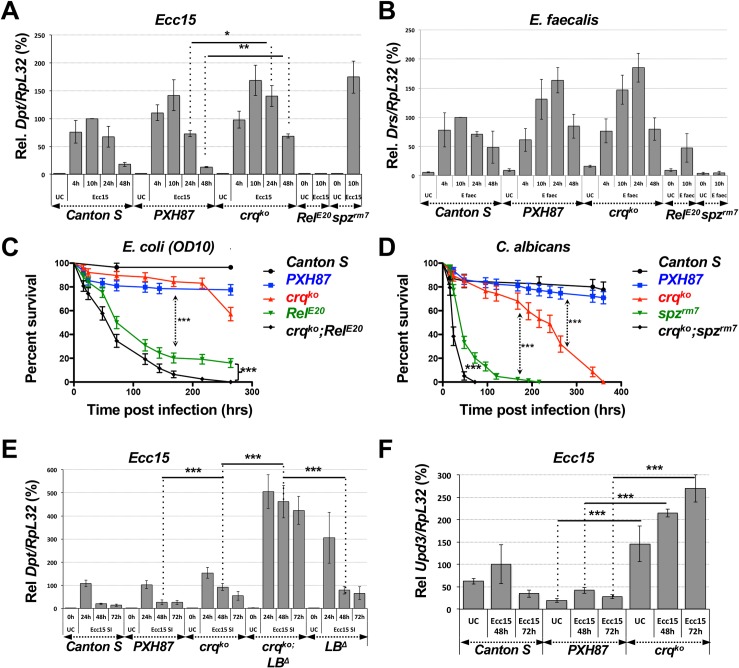
Crq acts in parallel to the Toll and Imd pathways. (**A**-**B**) Relative levels (in %) of *Dpt* mRNA expression (normalized against *RpL32*) compared to *Canton S* at 10hrs after *Ecc15* challenge, as determined by RT-qPCR on extracts of UC *Canton S*, *PXH87*, *crq* ^*ko*^, *Rel* ^*E20*^ and *spz* ^*rm7*^ mutant flies, or at 4, 10, 24 or 48hrs after pricking with *Ecc15* (**A**) or *E*. *faecalis* bacteria (**B**). Mean values of at least 3 repeats are represented ±SE. *p<0.05 ***p<0.001 (Student’s T-test). (**C**-**D**) Survival curves (in %) over time of *Canton S*, *PXH87*, *crq* ^*ko*^, *Rel* ^*E20*^ and *crq* ^*ko*^;*Rel* ^*E20*^ double mutant flies after septic injury with *E*. *coli* (**C**), or of *Canton S*, *PXH87*, *crq* ^*ko*^, *spz* ^*rm7*^ and *crq* ^*ko*^; *spz* ^*rm7*^ double mutant flies after septic injury with *C*. *albicans* (**D**). Curves represent average survivals ±SE. **p<0.01 ***p<0.001 in a log rank test. (**E**) Relative levels (in %) of *Dpt* mRNA expression (normalized against *RpL32*) compared to *Canton S* flies at 24hrs after *Ecc15* challenge, as determined by RT-qPCR on extracts of UC *Canton S*, *PXH87*, *crq* ^*ko*^, *PGRP-LB* ^*Δ*^ and *crq* ^*ko*^; *PGRP-LB* ^*Δ*^ double mutant flies, or at 24, 48 or 72hrs after challenge with *Ecc15*. Mean values of at least 3 repeats are represented ±SE. ***p<0.001 (Student’s T-test). (**F**) Relative levels (in %) of *upd3* mRNA expression (normalized against *RpL32*) compared to *Canton S* at 48hrs after *Ecc15* challenge, as determined by RT-qPCR on extracts of UC *Canton S*, *PXH87* and *crq* ^*ko*^ mutant flies, or at 48 and 72hrs after challenge with *Ecc15*. Mean values of at least 3 repeats are represented ±SE. ***p<0.001 (Student’s T-test).

Phagocytosis has been proposed as a key step to initiate AMP production [21]. To assess the effect of mutating *crq* on AMP production downstream of both the Imd and Toll pathways, we next quantified the expression of Diptericin (*Dpt*) and Drosomycin (*Drs*)-encoding genes by RT-qPCR after *Ecc15* or *E*. *faecalis* infections (**Fig 5A and 5B**). As previously reported, septic injury of control flies with *Ecc15* induced *Dpt* expression, which peaked at 10 hpi and returned to near-basal levels within 48 hpi (**Fig 5A)**[7]. In *crq* ^*ko*^ flies, *Dpt* expression was 2-fold higher than in control flies at 10hrs post-infection and failed to return to basal levels within 48hrs (**Fig 5A**). In contrast, there was no significant difference in *Drs* induction between control and *crq* ^*ko*^ flies after *E*. *faecalis* inoculation (**Fig 5B**). Survival curves indicated that *crq* ^*ko*^ flies were less susceptible to a non-pathogenic *E*. *coli* infection than *Rel* ^*E20*^ flies, while double mutants for *crq* ^*ko*^ and *Rel* ^*E20*^ were statistically more susceptible than *Rel* ^*E20*^ or *crq* ^*ko*^ mutants alone (**Fig 5C**). The extreme sensitivity of *Rel* ^*E20*^ flies to infection with pathogenic bacteria prevented us from carrying out these experiments with *Ecc15*. Instead, we inoculated the flies with 20 times fewer *E*. *coli* than previously used in Fig 2. Similarly, *crq* ^*ko*^ and *spz* ^*rm7*^ double mutants were also statistically more susceptible to *C*. *albicans* infection than the *spz* ^*rm7*^ or *crq* ^*ko*^ mutants alone (**Fig 5D**). Therefore, *crq* is not required for the induction of AMPs and acts in parallel to the Toll and Imd pathways.

These results suggested that aberrant phagocytosis in *crq* ^*ko*^ flies can result in enhanced and persistent Imd pathway activation. Multiple negative regulators of the Imd pathway help maintain immune homeostasis. For example, Peptidoglycan Recognition Proteins (PGRPs) with amidase activity, such as PGRP-LB, degrade immunostimulatory molecules [15]. Thus, we next assessed *Dpt* expression levels by RT-qPCR in single and double *PGRP-LB* ^*Δ*^ and *crq* ^*ko*^ mutants upon *Ecc15* infection. Single *crq* ^*ko*^ and *PGRP-LB* ^*Δ*^ mutants expressed statistically higher levels of *Dpt* than *Cs* and *PXH87* controls at 48hrs (**Fig 5E**). The *Dpt* expression resolved back to basal levels within 72hrs post infection in control flies, but remained high in single *PGRP-LB* ^*Δ*^ or *crq* ^*ko*^ mutants despite a steady decline in its expression (**Fig 5E**). Moreover, double mutants for *crq* ^*ko*^ and *PGRP-LB* ^*Δ*^ expressed *Dpt* at levels 5-fold higher than controls at 24hrs post-infection, and levels remained high at 48 and 72hrs (**Fig 5E**). These results demonstrate the critical interplay between phagocytosis and negative regulators of the immune system to achieve proper resolution of AMP expression upon systemic infection.

Plasmatocytes are also a major source of cytokine production upon systemic infection. Upd3, the *Drosophila* analogue of IL-6, can induce the JAK-STAT pathway, which regulates the systemic immune response and metabolic homeostasis in the fat body, as well as gut homeostasis [6,22,69,70]. Using RT-qPCR, we asked whether *crq* is required for *upd3* expression upon *Ecc15* infection. Control flies displayed a small and temporary induction of *upd3* expression that resolved within 72hrs (**Fig 5F**). In contrast, UC and *Ecc15*-challenged *crq* ^*ko*^ flies showed a 1.5-fold stronger induction of *upd3* expression, which further increased over 72hrs (**Fig 5F**). Thus *crq* is not required to induce *upd3* expression, but *crq* mutation results in enhanced and continuously increasing *upd3* expression. Altogether, these results demonstrate that *crq* is required for bacterial clearance and mutation of *crq* alters the resolution of AMPs and Upd3 cytokine production.

### *croquemort* mutant flies have a short lifespan with early gut dysplasia

**Fig 6:**
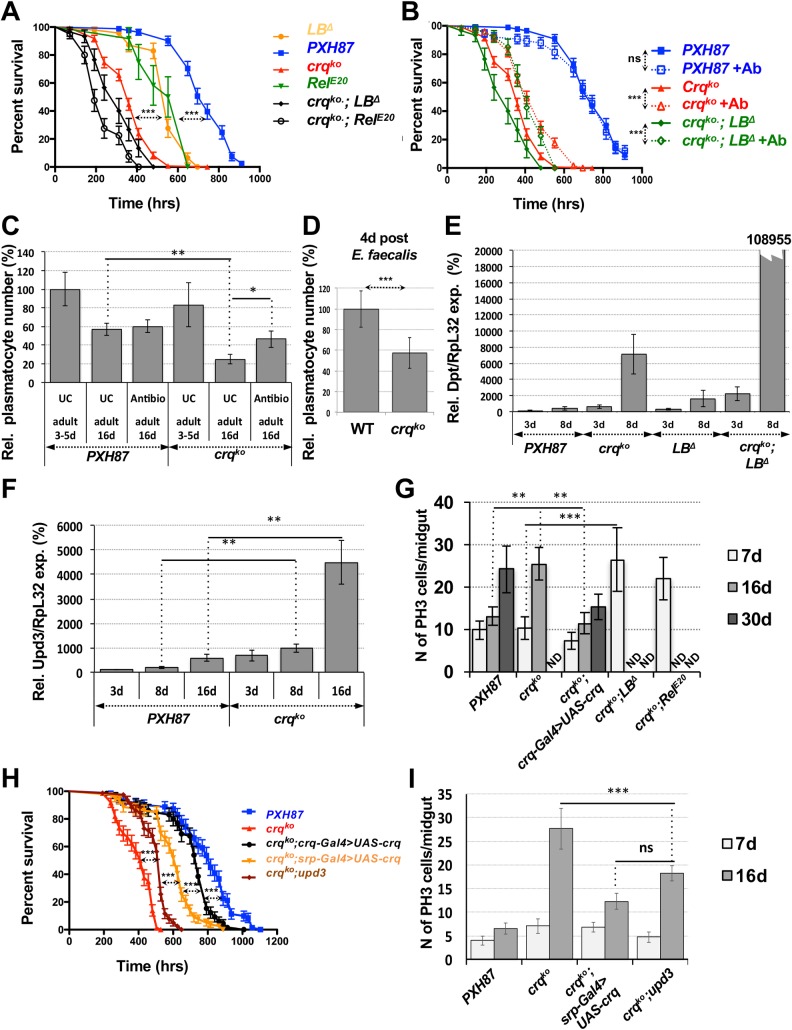
*crq* knock-out flies show early midgut dysplasia and a shorter lifespan. **(A)** Survival curves (in %) over time of *PXH87*, *crq* ^*ko*^, *Rel* ^*E20*^, *PGRP-LB* ^*Δ*^, *crq* ^*ko*^ *;Rel* ^*E20*^ and *crq* ^*ko*^ *;PGRP-LB* ^*Δ*^ double mutant flies. (**B**) Survival curves (in %) over time of *PXH87*, *crq* ^*ko*^, and *crq* ^*ko*^ *;PGRP-LB* ^*Δ*^ double mutant flies raised on conventional or antibiotics-supplemented medium. Curves represent average survivals ±SE. **p<0.01 ***p<0.001 in a log rank test. (**C**) Hemocyte counts of 3–5 and 16 days old *PXH87* and *crq* ^*ko*^ adult flies raised in UC conditions on conventional or antibiotics-supplemented medium. Mean values of at least 3 repeats are represented ±SE. *p<0.05 **p<0.01. (**D**) Relative number (in %) of plasmatocytes in *crq* ^*ko*^ flies compared to WT flies (normalized at 100%) 4 days post *E*. *faecalis* infection. Mean values of at least 3 repeats are represented ± SE. *p<0.05 ***p<0.001 (Student’s T-test). (**E**) Relative levels (in %) of *Dpt* mRNA expression (normalized against *RpL32*) compared to 3 days old *PXH87* flies, as determined by RT-qPCR on extracts of UC 3 or 8-days old *PXH87*, *crq* ^*ko*^, *PGRP-LB* ^*Δ*^, *and crq* ^*ko*^ *; PGRP-LB* ^*Δ*^ double mutant flies. (**F**) Relative levels (in %) of *upd3* mRNA expression levels (normalized against *RpL32*) compared to 3 days old *PXH87* flies, as determined by RT-qPCR on extracts of UC 3, 8 and 16 days old *PXH87* and *crq* ^*ko*^ flies. (**G**) Number of mitotic PH3 positive cells per midgut of *PXH87*, *crq* ^*ko*^ and rescue flies, and of *crq* ^*ko*^;*PGRP-L*B^*Δ*^ and *crq* ^*ko*^;*Rel* ^*E20*^ double mutant flies. **(H)** Survival curves (in %) over time of *PXH87*, *crq* ^*ko*^, *upd3;crq* ^*ko*^ double mutant flies, as well as *crq-Gal4* and *srp-Gal4* rescue flies. (**I**) Number of mitotic PH3-positive cells per midgut of *PXH87*, *crq* ^*ko*^, *upd3;crq* ^*ko*^ double mutants and hemocyte-specific rescue flies. Mean values of at least 3 repeats are represented ±SE. **p<0.01 ***p<0.001.

*PGRP-LB* ^*Δ*^ and *Rel* ^*E20*^ mutants all die prematurely, within about 696 hrs (29 days) of age, when compared to wild-type (p<0.0001) and *PXH87* (p<0.0001) control flies, which die after about 912 hrs (37 days) on conventional food at 29°C [15] **(Fig 6A)**. *crq* ^*ko*^ flies died on average within 552 hrs (23 days), considerably earlier than *Rel* ^*E*20^ and *PGRP-LB* ^*Δ*^ mutants (p<0.0001). Double c*rq* ^*ko*^ and *PGRP-LB* ^*Δ*^ or *crq* ^*ko*^ and *Rel* ^*E20*^ mutants died within about 480 hrs (20 days) and 408 hrs (17 days) of age, respectively (**Fig 6A**). Antibiotic treatment partially rescued these phenotypes, as the lifespan of *crq* ^*ko*^ flies and the double mutants increased significantly (p<0.0001) (**Fig 6B**). To ask whether the premature aging of *crq* ^*ko*^ flies might correlate with a loss of immune cells or their function, we estimated the number of plasmatocytes present in control and *crq* ^*ko*^ flies using the *eater-nlsGFP* reporter (**Fig 6C**). As previously reported [56,57], the number of plasmatocytes was decreased by about 40% in 16-day-old control flies (**Fig 6C**), while similarly aged *crq* ^*ko*^ flies had lost 80% of their plasmatocytes (**Fig 6C**). Treatment with antibiotics rescued this *crq* ^*ko*^ phenotype but had no effect on the plasmatocyte counts of control flies. *crq* ^*ko*^ flies also lost about 40% of their plasmatocytes at 4 days post-*E*. *faecalis* infection when compared to similarly challenged wild-type controls (**Fig 6D**). This loss of *crq* ^*ko*^ hemocytes may be a consequence of accumulation of undigested bacteria inside their phagosomes. Thus, *crq* is required for plasmatocytes to survive innocuous or pathogenic bacterial infection.

*Dpt* expression in wild-type and *PXH87* flies is relatively low and stable over the first 8 days of their lives and increases as flies age [71] **(Fig 6E)**. Strikingly, *Dpt* expression was 70-fold higher in 8-day-old *crq* ^*ko*^ flies and nearly 1,100-fold higher in the double mutants for *crq* ^*ko*^ *and PGRP-LB* ^*Δ*^ compared to controls (**Fig 6E**). Thus, the Imd pathway is strongly up-regulated early on in the life of these mutant flies, even in the absence of infection. This points to a role for Crq in phagocytosis and in maintaining immune homeostasis. Likewise, *upd3* expression steadily increased as *PXH87* flies aged, and it was further enhanced by nearly 10-fold in 8- and 16-day-old *crq* ^*ko*^ flies (**Fig 6F**). Antibiotic treatment partially rescued the levels of *Dpt* expression in *crq* ^*ko*^ flies (**S6A Fig**), arguing that the hyper-activation of the Imd pathway in these flies results from their inability to control environmental microbes. To address this, we plated fly extracts on both LB (on which most pathogens can grow) and MRS (on which most *Drosophila* microbiota can grow) agar plates and quantified the resulting CFUs (**S6B Fig**). In line with previous studies, the CFUs obtained from 2 week-old control flies were in the range of 2,000 per fly (**S6B Fig**) [72–74]. Significantly fewer CFUs were recovered from *PGRP-LB* ^*Δ*^ mutants, while both *crq* ^*ko*^ and *Rel* ^*E20*^ extracts showed a 10-fold increase. Double mutants for *crq* ^*ko*^ and *Rel* ^*E20*^ had 50-fold more CFUs than controls (**S6B Fig**). Altogether, these results demonstrate that Crq and the Imd pathway act in parallel and are required for the management of environmental microbes.

Elevated levels of Upd3 are associated with midgut hyperplasia in aging flies [72,75]. In addition, loss of gut barrier integrity leads to early death in a microbiota-dependent manner [76,77]. Because 8-day-old *crq* ^*ko*^ flies expressed high levels of *upd3*, we asked whether they also displayed premature gut hyperplasia by looking at the number of mitotic PH3-positive intestinal stem cells of their midgut. While *PXH87* and *crq* ^*ko*^ flies did not show any signs of midgut hyperplasia at day 7, midguts of 16 day-old *crq* ^*ko*^ flies had a 2-fold increase in PH3-positive cells compared to that of similarly aged controls (p = 0.0109) (**Fig 6G**). This phenotype was completely rescued in *crq* ^*ko*^ *; crq-Gal4>UAS-crq* flies (**Fig 6G**). The double mutants for *crq* ^*ko*^ and *PGRP-LB* ^*Δ*^ or for *crq* ^*ko*^ and *Rel* ^*E20*^ showed even higher levels of intestinal stem cell proliferation than controls (p = 0.03) and did so more prematurely (already in 7-day-old flies) (**Fig 6G**). The premature increase in midgut stem cell proliferation was partially dependent on Upd3, as *upd3;crq* ^*ko*^ double mutant flies had significantly less mitotic cells (p = 0.04) and lived longer than *crq* ^*ko*^ flies (p<0.0001) (**Fig 6H and 6I**). However, the lifespan of *upd3;crq* ^*ko*^ double mutants flies was still shorter than that of *PXH87* flies (p<0.0001), suggesting that additional mechanisms play a role in the shortened lifespan of *crq* ^*ko*^ flies. We further asked whether *crq* is required in hemocytes to maintain intestinal homeostasis. Hemocyte-specific re-expression of *crq* led to a strong rescue of lifespan compared to *crq* ^*ko*^ flies (p<0.0001) but not to the levels of PXH87 flies (p = 0.0462) and to a partial rescue of midgut hyperplasia in 16-day-old flies (p = 0.003 for *crq* ^*ko*^ vs rescue and p = 0.0123 for rescue vs PXH87) (**Fig 6H and 6I**). Altogether, these results indicate that flies lacking *crq* display chronically elevated expression of *upd3* that triggers early midgut hyperplasia and promotes premature death.

## Discussion

Our study shows that Crq is required for the engulfment of microbes by plasmatocytes and their clearance, and that the mild immune deficiency due to *crq* mutation is associated with increased susceptibility to infection, defects in immune homeostasis, gut hyperplasia, and decreased lifespan (**S7 Fig**). We have also re-confirmed a role for *crq* in apoptotic cell clearance, although the phagocytosis defect of *crq* ^*ko*^ plasmatocytes is less severe than what had been previously observed with two lethal *crq* deficiency mutants, *Df(2L)al* and *Df(2L)XW88* [35]. A possible explanation is that these deficiencies may have deleted at least one other gene required for apoptotic cell clearance. Additionally, morphological defects associated with secondary mutations could have exacerbated the *crq* phagocytosis defect by preventing efficient plasmatocyte migration to apoptotic cells. These same deficiency mutants had been assessed qualitatively for phagocytosis of bacteria by injecting embryos with *E*. *coli* or *S*. *aureus*; their plasmatocytes had no obvious defect in their ability to engulf these bacteria [35]. However, a role for *crq* in phagocytosis of *S*. *aureus*, but not that of *E*. *coli*, was subsequently proposed based on S2 cell phagocytosis assays following knock-down of *crq* by RNAi [41]. Here, we show that *crq* is required *in vivo* for uptake and phagosome maturation of both *S*. *aureus* and *E*. *coli*. A simple explanation of this discrepancy with *E*. *coli* could be that knocking down *crq* by RNAi is not sufficient to affect its role in *E*. *coli* phagocytosis (but sufficient to affect its role in *S*. *aureus* phagocytosis), and that completely abrogating *crq* expression by *in vivo* knock-out leads to a stronger phenotype with both bacteria. Our *in vivo* data in *crq* ^ko^ flies further demonstrate that *crq* is required to resist multiple microbial infections, such as *Ecc15*, *E*. *faecalis*, *B*. *bassiana*, and *C*. *albicans*. These data therefore argue that *crq* plays a more general role in microbial phagocytosis than was previously anticipated. Our previous experiments to test whether *crq* is required for bacterial phagocytosis in embryos were qualitative rather than quantitative, and did not allow us to identify a role for *crq* at that stage [53]. In contrast, the experiments we now report in adult *crq* ^*ko*^ flies are quantitative and allowed us to identify a delay in phagocytosis, followed by a defect in bacterial clearance in *crq* ^ko^ hemocytes. A possible explanation for this discrepancy would be that hemocytes may differ in their expression profile, behavior, and phagocytic ability at various developmental stages due to differences in their microenvironment and/or sensitivity to stimuli. Accordingly, it has recently been shown that the phagocytic activity of embryonic hemocytes acts as a priming mechanism, increasing the ability of primed cells to phagocytose bacteria at later stages [78]. It is therefore possible that embryonic, larval and adult hemocytes display very different levels of priming and bacterial phagocytic activity, and that *crq* is required mostly in larval/adult bacterial phagocytosis. Alternatively, a potential defect in phagocytosis of bacteria by embryonic hemocytes of the *crq* deficiencies may have been suppressed by the deletion of (an)other gene(s) in that genomic region.

Because the immune competence of hemocytes varies during development [50,79,80], we were prompted to re-examine the potential role for *crq* in innate immunity by knocking it out. Here, we show that Crq is a major plasmatocyte marker at all developmental stages of the fly. We have found that *crq* ^*ko*^ flies are homozygous viable, but short-lived, and can hardly be maintained as a homozygous stock in a non-sterile environment; *crq* ^*ko*^ pupae become susceptible to environmental bacteria and their microbiota during pupariation. In a recent study, Arefin and colleagues induced the pro-apoptotic genes *hid* or *Grim* in plamatocytes and crystal cells using the *hml-gal4* driver (Hml-apo) and observed a similar pupal lethality, but also associated with an induction of lamellocyte differentiation, and the apparition of melanotic tumors of hemocyte origin [81]. The authors therefore concluded that the death of hemocytes triggered lamellocyte accumulation and melanotic tumor phenotypes [81]. In contrast, we did not observe any obvious melanotic tumors in *crq* ^*ko*^ flies, despite observing a loss of hemocytes in aging *crq* ^*ko*^ flies (**Fig 6C**) and *crq* ^*ko*^ flies subjected to *Ecc15* infection (**Fig 6D**). One possible explanation is that hemocytes do not die of apoptosis in *crq* ^*ko*^ flies, but of a distinct mechanism. Alternatively, *crq* mutation could affect more hemocytes than Hml-apo flies, as *crq* is expressed in all plasmatocytes, while *Hml* is only expressed in 72.4% of all plasmatocytes expressing *crq* (from **Fig 1C)**. Thus the 27.6% of non-Hml plasmatocytes (thus non induced for apoptosis, which is *hml-Gal4* dependent [81]) may respond to the death of the other plasmatocytes by inducing a signal that triggers the induction of lamellocytes and the subsequent formation of melanotic tumors. Considering the role of *crq* in apoptotic cell clearance, this signal may require a functional *crq*, which could explain why *crq* ^*ko*^ flies do not develop melanotic tumors. Strikingly, in the Arefin study, as well as in previous studies, targeted ablation of plasmatocytes also made resulting ‘hemoless’ pupae more susceptible to environmental microbes [23,24,81]. Extensive tissue remodeling takes place at pupariation, and plasmatocytes are essential to remove dying cells, debris, and bacteria. Thus, it was argued that this increased susceptibility was likely due to environmental bacteria invading the body cavity after disruption of the gut [82]. In addition, it was found that the gut microbiome of Hml-apo flies could influence pupal lethality, as the eclosure rate of Hml-apo flies varied depending on the quality of the food they were reared on [81]. Accordingly, our rescue of the *crq* ^*ko*^ pupal lethality with antibiotics demonstrates that their premature aging and death are indeed due to infection by normally innocuous environmental bacteria. Altogether, these data suggest that phagocytes and *crq* are important actors regulating the interaction between a host and its microbiome.

Hosts use both resistance and tolerance mechanisms to withstand infection and survive a specific dose of microbes [65,83]. *crq* ^*ko*^ flies exhibit a shorter lifespan when compared to control flies, but they are equally tolerant to aseptic wounds and infections. The *crq* ^*ko*^ flies are less resistant to infection, as *crq* is required to promote efficient microbial phagocytosis. *crq* ^*ko*^ plasmatocytes can still engulf bacteria, albeit at a lower efficiency than their controls. Our data also demonstrate that *crq* plays a major role in phagosome maturation during bacterial clearance. This is in agreement with a recent study showing that *crq* promotes phagosome maturation during the clearance of neuronal debris by epithelial cells [36]. Thus, *crq* is required at several stages of phagocytosis. Similar observations have been made for the *C*. *elegans* Ced-1 receptor and for Drpr, as both promote engulfment of apoptotic corpses and their degradation in mature phagosomes [84,85].

‘Hemoless’, Hml-apo and *crq* ^*ko*^ flies are all more susceptible to environmental microbes and their microbiota. While it is not known whether mutants of *eater*, which encodes a phagocytic receptor for bacteria but does not play a role in phagosome maturation, are more susceptible to environmental microbes during pupariation, both *eater* mutants and ‘hemoless’ flies showed either decreased or unaffected systemic responses [23,24,26]. Hml-apo larvae however, showed an upregulation in Toll-dependent constitutive *Drs* mRNA levels whereas *Dpt* expression was suppressed [81]. In contrast, *crq* ^*ko*^ flies showed no significant difference in constitutive or infection induced expression of *Drs*, but showed an increased expression of *Dpt* with age, and infection induced an increased and chronic expression of *Dpt*. Altogether our results argue that phagosome maturation defects in *crq* ^*ko*^ flies lead to persistence of bacteria and thus to an increased and persistent systemic immune response via the Imd pathway. Such defects in phagosome maturation are not present in hemocyte ablation experiments, which could explain different outcomes for the host immunity and survival.

We have found that Crq acts in parallel to the Toll and Imd pathways. In the mealworm *Tenebrio molitor*, hemocytes and cytotoxic enzymatic cascades eliminate most bacteria early during infection, and AMPs are required to eliminate persisting bacteria [86]. These data suggest that AMPs act in parallel with hemocytes to fight infections. We have also found that *crq* ^*ko*^ flies are more susceptible to infection with *S*. *aureus* than wild-type and Toll pathway-deficient flies. These results are consistent with *S*. *aureus* infection being mainly resolved via phagocytosis and Crq having a major role in this process. Surprisingly, we have observed the opposite for infection with other Gram-negative or positive bacteria and fungi. *Drosophila* mutants for AMP production were more susceptible to infection than *crq* ^*ko*^ flies, arguing that AMPs are critical to eliminate the bulk of pathogens. Indeed, *crq* (thus phagocytosis) is not essential for *Ecc15* elimination, but accelerates bacterial clearance. Our results also suggest that the defects in phagosome maturation may allow some bacteria to persist and grow within hemocytes, where they are hidden from systemic AMPs. Thus, depending on the microbe, humoral and cellular immune responses can act at distinct stages of infection. In this context, phagocytosis acts as a main defense mechanism against pathogens that may escape AMPs or modulate their production.

Chronic activation of immune pathways can be detrimental to organismal health [13–15]. In *Drosophila*, multiple negative regulators of the Imd pathway, including PGRP-LB, act in concert to maintain immune homeostasis [14–16]. We have observed that *crq* ^*ko*^ flies sustain high production levels of the AMP Dpt and the cytokine Upd3, demonstrating that defects in phagocyte function can lead to chronic immune activation. Notably, the level of *Dpt* expression induced by activation of the Imd pathway in unchallenged conditions is stronger in *crq* ^*ko*^ flies than was previously observed in mutants of three negative regulators of the Imd pathway, namely *pirk* ^*EY*^, *PGRP-SC* ^*Δ*^, and *PGRP-LB* ^*Δ*^ [15], and over 1,000-fold higher in *PGRP-LB* ^*Δ*^, *crq* ^*ko*^ double mutants. This is despite the persistence of only a few hundred bacteria in these mutants. This phenotype may be due solely to the accumulation of these persistent bacteria, or Crq may also function in plasmatocytes to remove immunostimulatory molecules from the hemolymph. Nonetheless, our study shows that plasmatocytes, Crq, and phagocytosis are all key factors in the immune response, and that losing *crq* induces a state of chronic immune induction.

The ability of a host to control microbes decreases with age, a phenomenon called immune senescence [71]. The causes of immune senescence remain elusive, but the loss of immune cells with age and a decline in their ability to phagocytose have been suggested [56,57]. Recent studies have argued that microbial dysbiosis and disruption in gut homeostasis contribute to early aging [76,77,87]. In addition, persistent activation of the JAK-STAT pathway in the gut has been linked to age-related decline in gut structure and function [88]. Aging *crq* ^*ko*^ flies lose a greater number of hemocytes than wild-type flies after infection, which may be the result of accumulating bacteria in these hemocytes in which phagosomes fail to mature. The premature death of *crq* ^*ko*^ flies could be partially rescued by the presence of antibiotics. This demonstrates that phagocytosis, and phagosome maturation in particular, plays a crucial role in managing the response to environmental microbes and potentially, the gut microbiota directly to promote normal aging. We have also found that chronic *upd3* expression in *crq* ^*ko*^ flies triggers premature midgut hyperplasia, which is known to alter host physiology and promote premature aging [72,76,89]. It has recently been proposed that plasmatocytes can influence gut homeostasis by secreting dpp ligands and modulating stem cell activity [90]. Our results reinforce the possibility of an interaction between plasmatocyte function and gut homeostasis, and suggests that cytokines derived from hemocytes can trigger cell responses in the gut. These results are also in agreement with a recent publication showing that Upd3 from hemocytes can trigger intestinal stem cell proliferation [69]. Altogether, these results demonstrate that the interaction between hemocytes and the gut tissue are central to host health, and our data demonstrate that phagocytic defects can be associated with chronic gut inflammation and aberrant intestinal stem cell turn-over. As gut aging and barrier integrity are in turn important to maintain bodily immune homeostasis [76], we propose the following model: in *crq* ^*ko*^ flies, plasmatocyte-derived cytokines accelerate gut aging promoting loss of gut homeostasis and microbial dysbiosis, with immune and plasmatocyte activation acting in a positive feedback loop (**S7 Fig**).

Collectively, our data show that Crq is essential in development and aging to protect against environmental microbes. Interestingly, the impact of mutating *crq* on host physiology is strikingly different from previously reported phagocytic receptor mutations. We speculate that this could be due to its dual role in uptake and phagosome maturation during phagocytosis. Crq is required for microbial elimination in parallel to the Toll and Imd pathways and acts to maintain immune homeostasis. This situation is surprisingly reminiscent of inflammatory disorders, such as Crohn’s disease, that result from primary defects in bacterial elimination and trigger chronic immune activation and disruption of gut homeostasis. Further characterization of the *crq* mutation in *Drosophila* will provide an interesting conceptual framework to understand auto-inflammatory diseases and their repercussions on immune homeostasis and host health.

## Materials and Methods

### Fly rearing, stocks, and mutant generation

All stocks were raised at 22°C on standard medium, unless otherwise specified. *Rel* ^*E20*^, *spz* ^*rm7*^, and *PGRP-LB* ^*Δ*^ stocks were described in [15,61,91]. The *crq* ^*ko*^ stock was generated by homologous recombination, which removed the majority of the *crq* open reading frame [36] and (**S1B Fig**).

### Bacterial strains, infection experiments, and antibiotic treatment

For bacterial infections, males or females were pricked in the thorax with a needle previously dipped in a concentrated pellet of the tested pathogen. The following bacterial or yeast strains were used at the indicated optical density (OD) taken at 600 nm: *Ecc15* (OD = 200), *E*. *coli* (OD = 200 and OD = 10), *E*. *faecalis* (OD = 5), *S*. *aureus* (OD = 0.5), *C*. *albicans* (OD = 200). For *B*. *bassiana* infection, flies were shaken in a petri dish with mature germinating *Beauveria* for spore coating. All infections and aging experiments were performed at 29°C. In antibiotic treatments, a cocktail of kanamycin, ampicillin, rifampicin, streptomycin, and spectinomycin (5mg/mL each) was added to the fly medium. Axenic stocks were generated as described in [72,73]. Survival experiments represent at least 3 independent repeats with 20 flies (60–100 flies tested). Survival was analyzed by a Log-rank test using the statistical programs R and Prism.

### Quantification of bacterial CFUs

Flies were individually homogenized in 500 μl of sterile PBS using bead beating with a tissue homogenizer (OPS Diagnostics). Dilutions of the homogenate were plated onto LB agar or MRS agar with a WASP II autoplate spiral plater (Microbiology International), incubated at 29°C, and the CFUs counted. Results were analyzed using a Krustal-Wallis test in R.

### Phagocytosis assays and plasmatocyte immunostaining

Flies were injected in their thorax with 69nl of pHrodo red or Alexa 488 bacteria (Life Technologies Inc.) using a nanoject injector (Drummond). The fluorescence within the abdomen of the flies was then imaged at 45min, 3hrs, and 5hrs post-injection with a Leica MZFLIII fluorescent microscope and DFC300 FX camera and quantified using Image J 2.0.0-rc-30/1.49s (NIH).

For *ex vivo* imaging, flies were injected with 46nl of PBS at 45min, 3hrs and 5hrs after infection to release all hemocytes, and 10 flies were bled on a lysine-coated slide by mechanically scraping their hemocytes onto a drop of PBS. Once settled for 10min on the slide, hemocytes were quickly dried and mounted with AF1 mounting solution (Citifluor Ltd). Slides were automatically scanned using a Zeiss LSM 700 confocal microscope, and the number of plasmatocytes and average fluorescence signal per plasmatocyte quantified.

For immunostaining, flies were bled as described above and the hemocytes fixed in a solution of PBS, Tween 0.1%, PFA 4% for 30min. The samples were incubated in PBT with 1% normal goat serum and Crq [53] and GFP antibodies (Roche) at 1:500 overnight at 4°C. Samples were washed at RT three times for 5min in PBS, incubated with the appropriate secondary antibodies at 1:1000 in PBT for 2hrs at RT, and washed three additional times in PBT. Samples were imaged with a Zeiss LSM 700 confocal microscope.

### RT-qPCR

Total RNA was extracted from pools of 20 flies per time point using TRIzol (Invitrogen). RNA was reverse-transcribed using Superscript II (Invitrogen), and the qPCR was performed using SYBR green (Quanta) in a Biorad instrument. Data represent the ratio or relative ratio (in %) of mRNA levels of the target gene (*crq*, *Dpt*, *Drs* or *upd3*) and that of a reference gene (*RpL32* also known as *rp49*). The primer sequences used in this study are provided in the supplementary material. All experiments were performed at least 3 times.

## Supporting Information

S1 Text Supplementary Material and Methods. (DOCX)

S1 Fig *crq* is required for apoptotic cell clearance but not hematopoiesis. (**A**) Schematic of wild-type (WT) versus *crq*-targeted allele in which most of the *crq* ORF was replaced by the FP-*mini-w+* cassette. (**B**) Apoptotic cell phagocytosis indices of control *PXH87* and *crq* ^*ko*^ homozygous plasmatocytes of stage 13 embryos. (**C**) Characterization of the bleeding technique showing the average number of plasmatocytes per field of view in relation to the number of larvae bled. (**D**) Relative number of melanized dots following heat shock-induced crystal cell lysis in wild-type control (WT) versus *crq* ^*ko*^ mutant larvae.
(TIF)

S2 Fig *crq* is required in females to survive infection. (**A-B**) Relative levels (in %) of *crq* mRNA expression (normalized against *RpL32*) as determined by RT-qPCR on extracts of flies after 12, 36 and 132 hrs post infection with *Ecc15* (**A**) or *E*. *Faecalis* (**B**). (**C**) Percent survival over time of *Canton S* and *PXH87* flies, *crq* ^*ko*^, *Rel* ^*E20*^ homozygous female flies upon septic injury with *Ecc15*. (**D**) Percent survival over time of *PXH87*, *Df(2L)BSC16*/CyO heterozygous, *crq* ^*ko*^ homozygous and *crq* ^*ko*^ */Df(2L)BSC16* trans-heterozygous male flies upon *Ecc15* septic injury. (E-**I**) Percent survival over time of *Canton S* and *PXH87* flies, *crq* ^*ko*^, *Rel* ^*E20*^ or *spz* ^*rm7*^ homozygous female flies upon septic injury with *E*. *coli* (**E**), *E*. *faecalis* (**F**) or *C*. *albicans* (**G**), after natural infection with *B*. *bassiana* (**H**) or after infection with *S*. *aureus* (**I**). Curves represent average survival ±SE. *p<0.05 **p<0.01 ***p<0.001 in a log rank test.
(TIF)

S3 Fig Rescue of *crq* expression ameliorates survival to infection. (**A**-**C**) Percent survival over time of *PXH87* control, *crq* ^*ko*^ homozygous mutant and *crq* ^*ko*^ *; crq-Gal4>UAS-crq* rescue flies upon septic injury with *Ecc15* (**A**) and *E*. *faecalis* (**B**), as well as upon natural infection with *B*. *bassiana* (**C**). (**D-G**) Percent survival over time of *PXH87* control, *crq* ^*ko*^ homozygous mutant, *crq* ^*ko*^ *; crq-Gal4>UAS-crq* and *crq* ^*ko*^ *; srp-Gal4>UAS-crq* rescue flies upon septic injury with *Ecc15* (**D**), *E coli* (OD200) (**E**), *E*. *faecalis* (**F**) *and C*. *albicans* (**G**). Curves represent average survival ±SE. *p<0.05 and ***p<0.0001 in a log rank test.
(TIF)

S4 Fig *crq* mutation does not alter host tolerance to infection. (**A**) % survival over time of *PXH87* control and *crq* ^*ko*^ homozygous flies with or without aseptic wound. In the shaded area are the survival curves of *crq* ^*ko*^ flies upon multiple infections from Fig 1. (**B**) The relationship between health and bacterial load (tolerance curve) is depicted here. A tolerance curve adopts a sigmoid shape, and we focus on the linear part of the relationship, where tolerance is represented by the slope of the regression health/load. (**C**, **D**) Tolerance graph of *PXH87* and *crq* ^*ko*^ flies given as the plot of regression between LT50 and the log number of injected bacteria for *Ecc15* (**C**) or *E*. *faecalis* (**D**) septic injury. (**E**) Tolerance graph for *PXH87* and *crq* ^*ko*^ flies given as the plot of regression of their survival at 3 timepoints post infection against the log number of *Ecc15* CFUs present at the same timepoint.
(TIF)

S5 Fig *crq* is required for bacterial phagocytosis. (**A**, **B**) Fluorescent images of abdomen of control *pXH87*, *crq* ^*ko*^ and *crq* ^*ko*^; *crq-Gal4 > UAS-crq* rescue flies at 3hrs after injection of Alexa488 *E*. *coli* and Alexa488 *S*. *aureus*, *respectively*. (**C**, **D**) 3D reconstruction and sections of confocal zeta-stacks scans of *eater-nls*::*GFP* hemocytes. In WT most hemocytes internalize rhodamine E. coli at 45min post injection. In *crq* ^*KO*^ flies, a number of hemocytes instead show contact with bacteria not fully internalized. (**E**, **F**) Fluorescent images of abdomen of control *PXH87*, *crq* ^*ko*^ and *crq* ^*ko*^; *crq-Gal4 > UAS-crq* rescue flies at 3hrs after injection of pHrodo red-*E*. *coli* and pHrodo red-*S*. *aureus*, *respectively*. (**G**, **H**) Quantification of average *E*. *coli* or *S*. *aureus* pHrodo red fluorescence present per fly abdomen in control *PXH87*, *crq* ^*ko*^ mutant and *crq* ^*ko*^ *;crq-Gal4>UAS-crq* rescue flies, respectively. * p<0.5; ** p<0.01. (**I**) Average GFP fluorescence per plasmatocyte of UC or *PXH87* and *crq* ^*ko*^ flies at 4 days post *Ecc15-GFP* injection.
(TIF)

S6 Fig *crq* is required to manage environmental microbes. (**A**) Relative percentage of *Dpt* mRNA expression (normalized against *RpL32*) in *PXH87* and *crq* ^*ko*^ flies raised on conventional or antibiotics-supplemented medium compared to UC 16 days-old *PXH87* flies raised on conventional medium. (B) Number of CFUs per fly of 14 days-old *PXH87*, *crq* ^*ko*^, *Rel* ^*E20*^, *PGRP-LB* single mutants and *crq* ^*ko*^ *; Rel* ^*E20*^ or *crq* ^*ko*^ *; PGRP-LB* double mutant flies. a, b, c represents statistical grouping.
(TIF)

S7 Fig Model for *crq* requirement in immunity. In absence of *crq*, phagocytic function is decreased and absence of phagosome maturation is associated with a defect in bacterial clearance. This mild immune-deficiency in turns triggers a chronic activation of immune pathways and cytokine production, potentially secondary to the decreased bacterial clearance. This hyperactive immune response includes the activation of the Toll and Imd pathways, and the induction of the cytokine Upd3. This chronic immune activation results in the induction of early midgut hyperplasia and promotes a decrease in lifespan.
(TIF)
