# Supplementary material for: The Drosophila CD36 Homologue croquemort Is Required to Maintain Immune and Gut Homeostasis during Development and Aging
Source: PLoS Pathog. 2016 Oct 25;12(10):e1005961. doi: 10.1371/journal.ppat.1005961 (PMC5079587; doi:10.1371/journal.ppat.1005961)
Supplement: S1 Text — (DOCX) [file ppat.1005961.s001.docx]

**Supporting Information**

**Supplementary Material and Methods**

**Primers used were as follows:**

crq forward, 5’ GTGGAATGAAGCCGGAGAAG 3’;

crq reverse, 5’ GATGCCAATTCGGAGGAGAG 3’;

Dpt forward, 5’ GCTGCGCAATCGCTTCTACT 3’

Dpt reverse, 5’ TGGTGGAGTGGGCTTCATG 3’;

Drs forward, 5’ CGTGAGAACCTTTTCCAATATGATG 3’;

Drs reverse, 5’ TCCCAGGACCACCAGCAT 3’;

RpL32 forward, 5’ GACGCTTCAAGGGACAGTATCTG 3’;

RpL32 reverse, 5’ AAACGCGGTTCTGCATGAG 3’;

Upd3 forward, 5’ GCGGGGAGGATGTACC 3’;

Upd3 reverse, 5’ GTCTTCATGGAATGAGCC 3’.
