# Supplementary figures and images for: The Drosophila CD36 Homologue croquemort Is Required to Maintain Immune and Gut Homeostasis during Development and Aging
Source: PLoS Pathog. 2016 Oct 25;12(10):e1005961. doi: 10.1371/journal.ppat.1005961 (PMC5079587; doi:10.1371/journal.ppat.1005961)

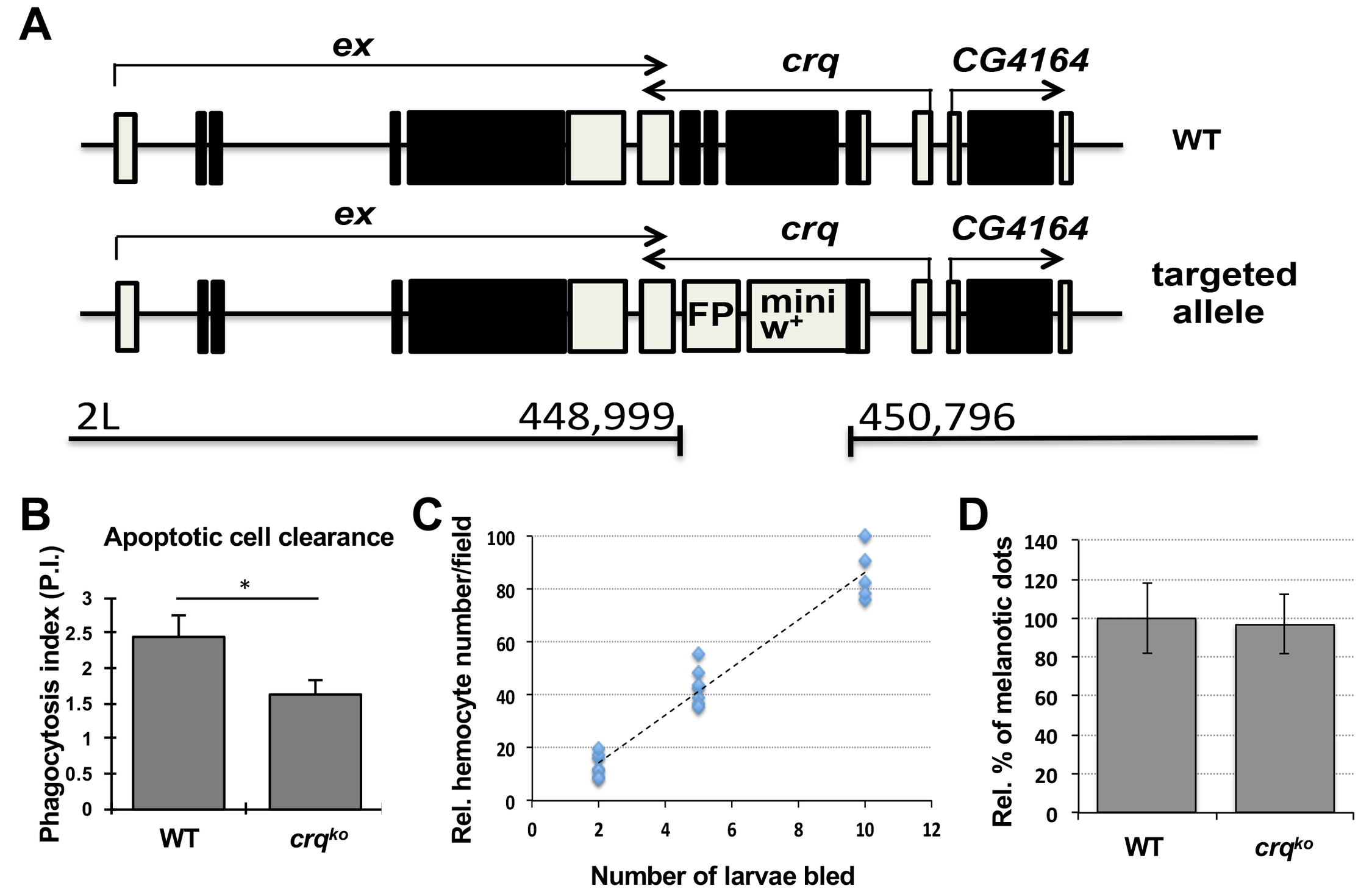

Supplement: S1 Fig — (A) Schematic of wild-type (WT) versus crq-targeted allele in which most of the crq ORF was replaced by the FP-mini-w+ cassette. (B) Apoptotic cell phagocytosis indices of control PXH87 and crq ko homozygous plasmatocytes of stage 13 embryos. (C) Characterization of the bleeding technique showing the average number of plasmatocytes per field of view in relation to the number of larvae bled. (D) Relative number of melanized dots following heat shock-induced crystal cell lysis in wild-type control (WT) versus crq ko mutant larvae. (TIF) [file ppat.1005961.s002.tif]

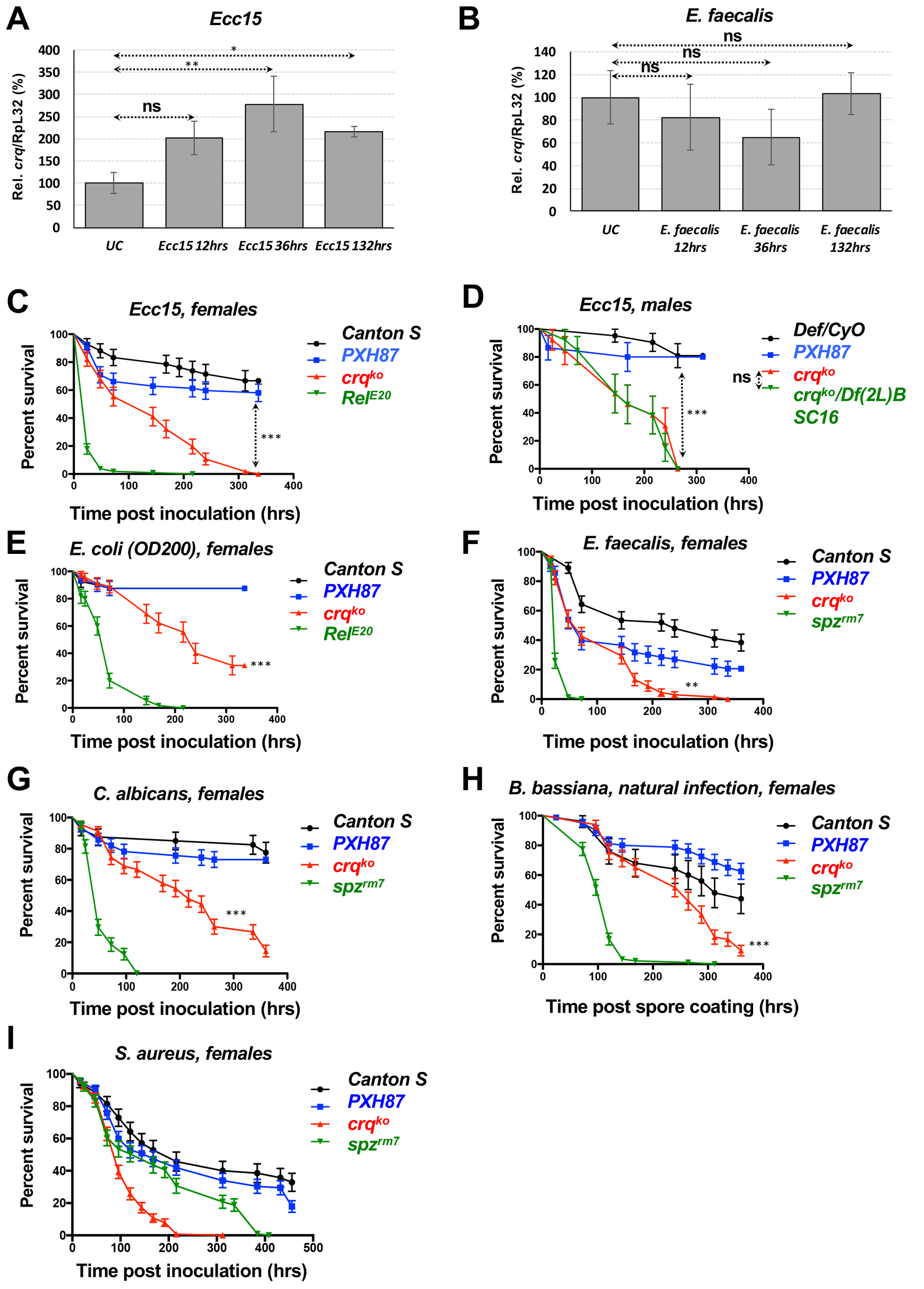

Supplement: S2 Fig — (A-B) Relative levels (in %) of crq mRNA expression (normalized against RpL32) as determined by RT-qPCR on extracts of flies after 12, 36 and 132 hrs post infection with Ecc15 (A) or E. Faecalis (B). (C) Percent survival over time of Canton S and PXH87 flies, crq ko, Rel E20 homozygous female flies upon septic injury with Ecc15. (D) Percent survival over time of PXH87, Df(2L)BSC16/CyO heterozygous, crq ko homozygous and crq ko /Df(2L)BSC16 trans-heterozygous male flies upon Ecc15 septic injury. (E-I) Percent survival over time of Canton S and PXH87 flies, crq ko, Rel E20 or spz rm7 homozygous female flies upon septic injury with E. coli (E), E. faecalis (F) or C. albicans (G), after natural infection with B. bassiana (H) or after infection with S. aureus (I). Curves represent average survival ±SE. *p<0.05 **p<0.01 ***p<0.001 in a log rank test. (TIF) [file ppat.1005961.s003.tif]

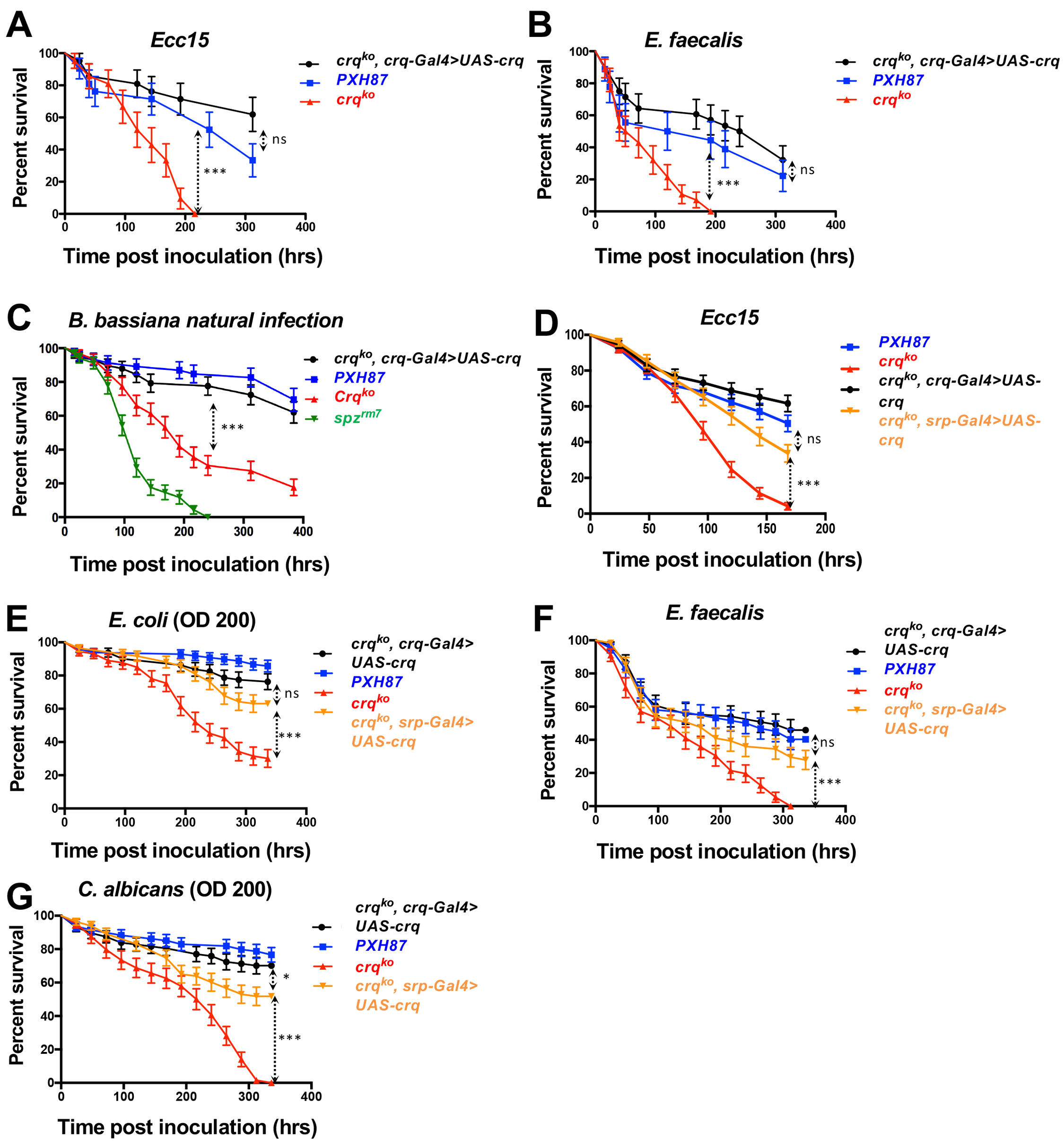

Supplement: S3 Fig — (A-C) Percent survival over time of PXH87 control, crq ko homozygous mutant and crq ko ; crq-Gal4>UAS-crq rescue flies upon septic injury with Ecc15 (A) and E. faecalis (B), as well as upon natural infection with B. bassiana (C). (D-G) Percent survival over time of PXH87 control, crq ko homozygous mutant, crq ko ; crq-Gal4>UAS-crq and crq ko ; srp-Gal4>UAS-crq rescue flies upon septic injury with Ecc15 (D), E coli (OD200) (E), E. faecalis (F) and C. albicans (G). Curves represent average survival ±SE. *p<0.05 and ***p<0.0001 in a log rank test. (TIF) [file ppat.1005961.s004.tif]

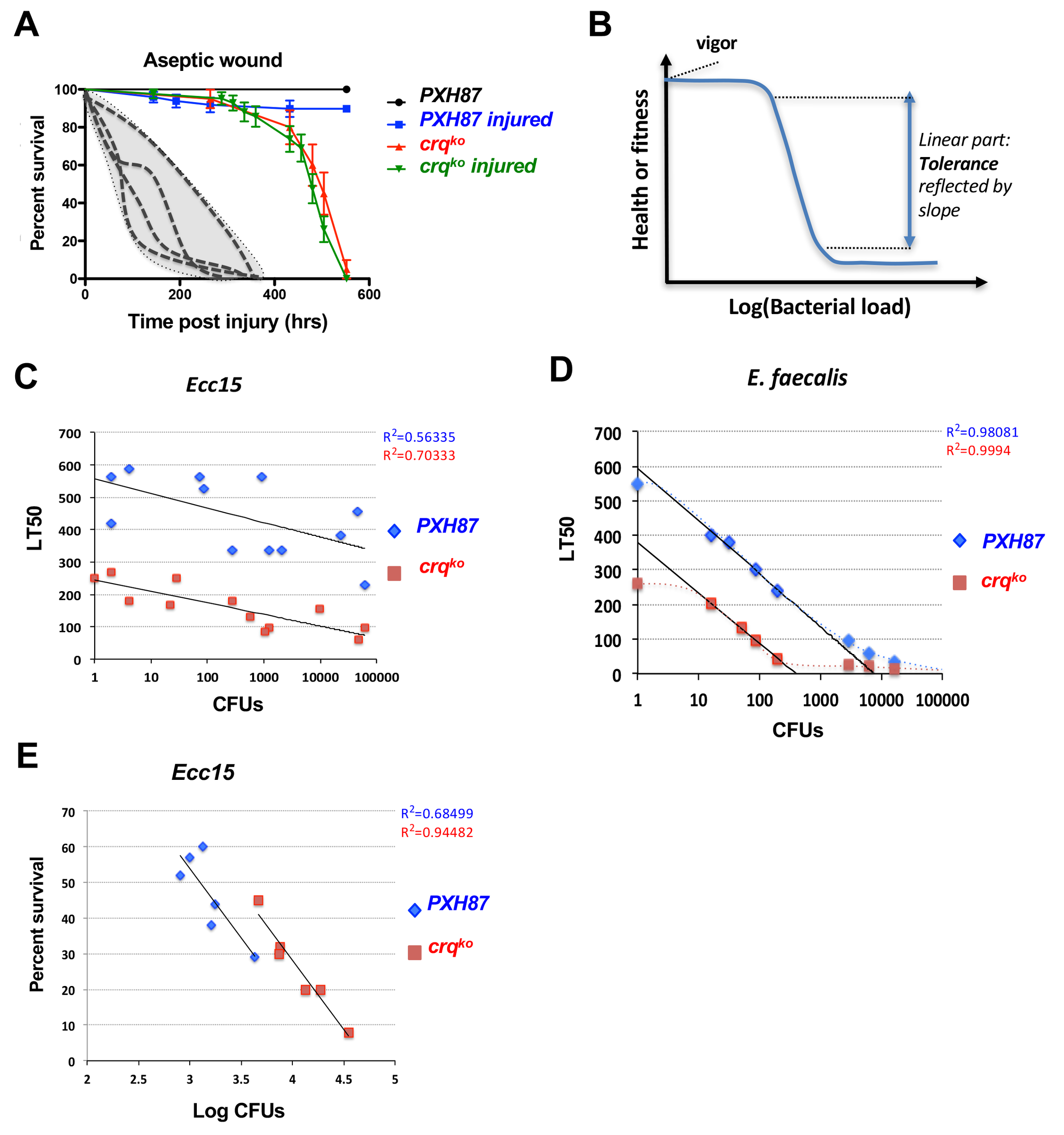

Supplement: S4 Fig — (A) % survival over time of PXH87 control and crq ko homozygous flies with or without aseptic wound. In the shaded area are the survival curves of crq ko flies upon multiple infections from Fig 1. (B) The relationship between health and bacterial load (tolerance curve) is depicted here. A tolerance curve adopts a sigmoid shape, and we focus on the linear part of the relationship, where tolerance is represented by the slope of the regression health/load. (C, D) Tolerance graph of PXH87 and crq ko flies given as the plot of regression between LT50 and the log number of injected bacteria for Ecc15 (C) or E. faecalis (D) septic injury. (E) Tolerance graph for PXH87 and crq ko flies given as the plot of regression of their survival at 3 timepoints post infection against the log number of Ecc15 CFUs present at the same timepoint. (TIF) [file ppat.1005961.s005.tif]

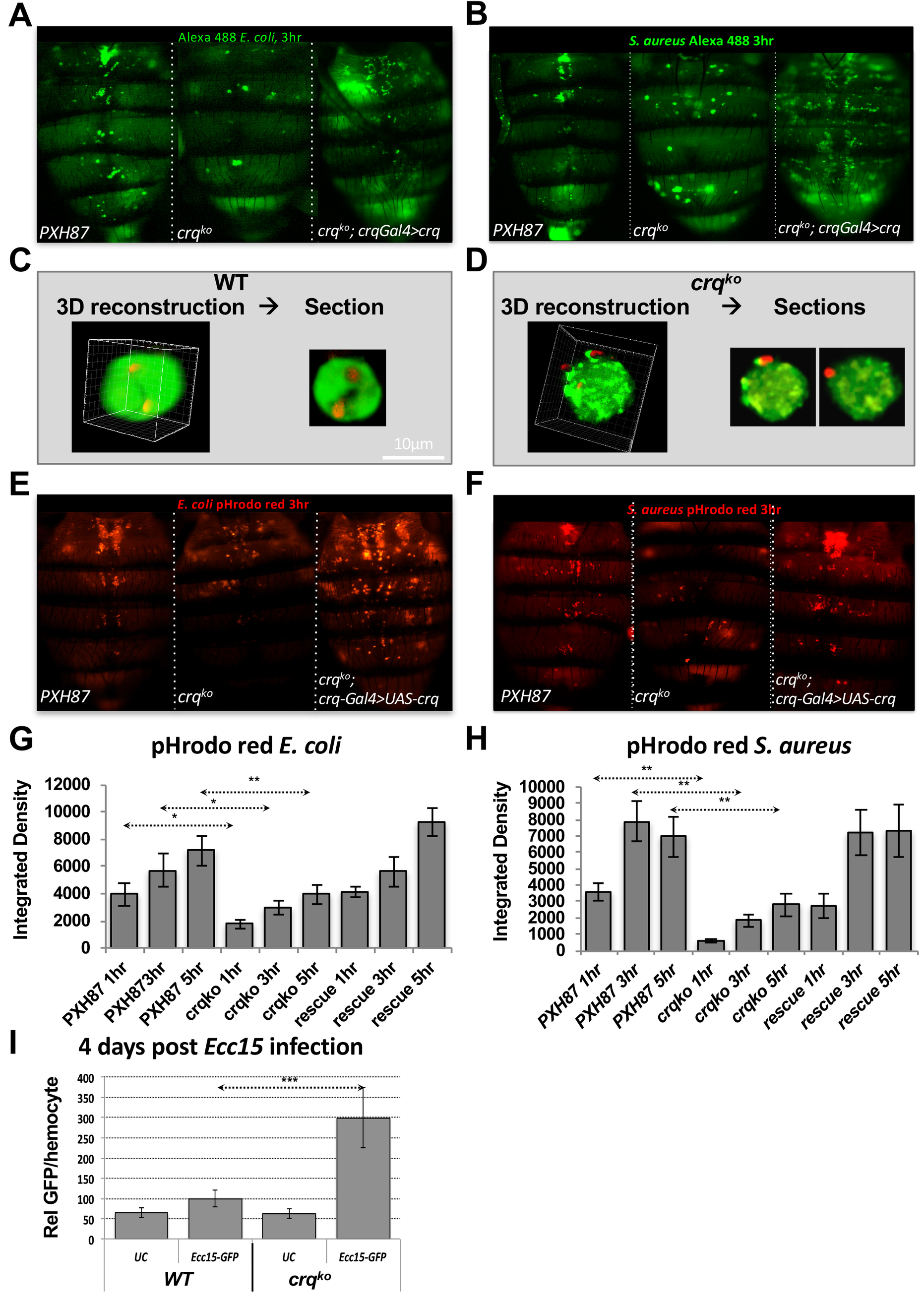

Supplement: S5 Fig — (A, B) Fluorescent images of abdomen of control pXH87, crq ko and crq ko; crq-Gal4 > UAS-crq rescue flies at 3hrs after injection of Alexa488 E. coli and Alexa488 S. aureus, respectively. (C, D) 3D reconstruction and sections of confocal zeta-stacks scans of eater-nls::GFP hemocytes. In WT most hemocytes internalize rhodamine E. coli at 45min post injection. In crq KO flies, a number of hemocytes instead show contact with bacteria not fully internalized. (E, F) Fluorescent images of abdomen of control PXH87, crq ko and crq ko; crq-Gal4 > UAS-crq rescue flies at 3hrs after injection of pHrodo red-E. coli and pHrodo red-S. aureus, respectively. (G, H) Quantification of average E. coli or S. aureus pHrodo red fluorescence present per fly abdomen in control PXH87, crq ko mutant and crq ko ;crq-Gal4>UAS-crq rescue flies, respectively. * p<0.5; ** p<0.01. (I) Average GFP fluorescence per plasmatocyte of UC or PXH87 and crq ko flies at 4 days post Ecc15-GFP injection. (TIF) [file ppat.1005961.s006.tif]

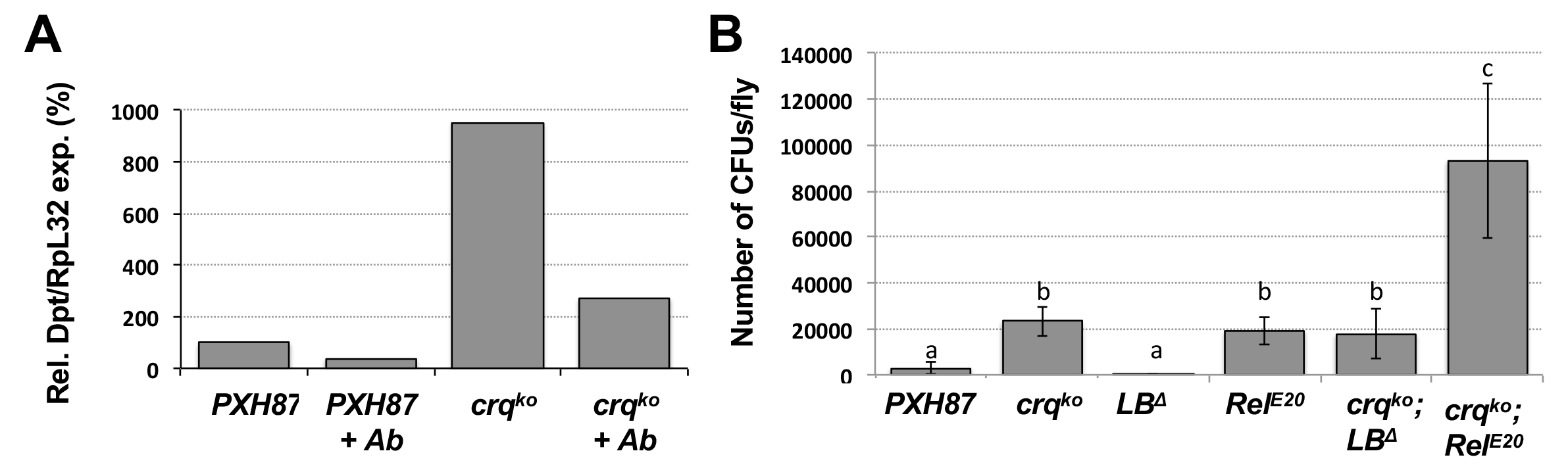

Supplement: S6 Fig — (A) Relative percentage of Dpt mRNA expression (normalized against RpL32) in PXH87 and crq ko flies raised on conventional or antibiotics-supplemented medium compared to UC 16 days-old PXH87 flies raised on conventional medium. (B) Number of CFUs per fly of 14 days-old PXH87, crq ko, Rel E20, PGRP-LB single mutants and crq ko ; Rel E20 or crq ko ; PGRP-LB double mutant flies. a, b, c represents statistical grouping. (TIF) [file ppat.1005961.s007.tif]

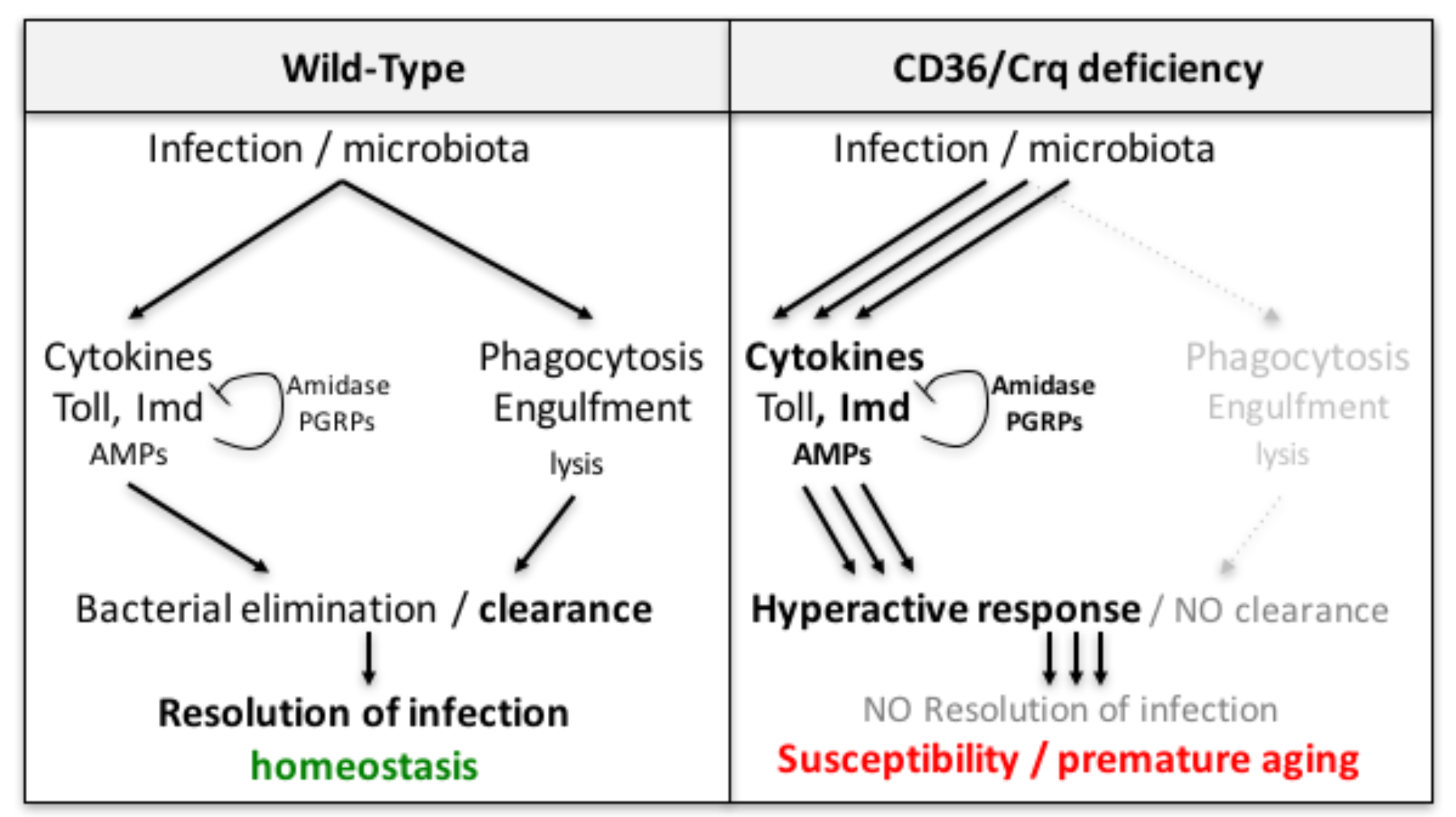

Supplement: S7 Fig — In absence of crq, phagocytic function is decreased and absence of phagosome maturation is associated with a defect in bacterial clearance. This mild immune-deficiency in turns triggers a chronic activation of immune pathways and cytokine production, potentially secondary to the decreased bacterial clearance. This hyperactive immune response includes the activation of the Toll and Imd pathways, and the induction of the cytokine Upd3. This chronic immune activation results in the induction of early midgut hyperplasia and promotes a decrease in lifespan. (TIF) [file ppat.1005961.s008.tif]
